# LncRNA IL21-AS1 facilitates tumour progression by enhancing CD24-induced phagocytosis inhibition and tumorigenesis in ovarian cancer

**DOI:** 10.1038/s41419-024-06704-8

**Published:** 2024-05-03

**Authors:** Jie Liu, Changsheng Yan, Shaohua Xu

**Affiliations:** 1 Department of Gynecology, Shanghai Key Laboratory of Maternal Fetal Medicine, Shanghai Institute of Maternal-Fetal Medicine and Gynecologic Oncology, Shanghai First Maternity and Infant Hospital, School of Medicine, Tongji University, Shanghai, China; 2 Department of Digestive Diseases, Huashan Hospital, Fudan University, Shanghai, China

**Keywords:** Cancer microenvironment, Immunoediting, Oncogenes

## Abstract

CD24 is overexpressed in various tumours and considered a regulator of cell migration, invasion, and proliferation. Recent studies have found that CD24 on ovarian cancer (OC) and triple-negative breast cancer cells interacts with the inhibitory receptor sialic-acid-binding Ig-like lectin 10 (Siglec-10) on tumour-associated macrophages (TAMs) to inhibit phagocytosis by macrophages. Because of its multiple roles in regulating the immune response and tumorigenesis, CD24 is a very promising therapeutic target. However, the regulatory mechanism of CD24 in OC remains unclear. Here, we found that the long noncoding RNA (lncRNA) IL21-AS1, which was upregulated in OC, inhibited macrophage-mediated phagocytosis and promoted OC cell proliferation and apoptosis inhibition. More importantly, after IL21-AS1 knockdown, a significant survival advantage was observed in mice engrafted with tumours. Mechanistically, we identified IL21-AS1 as a hypoxia-induced lncRNA. Moreover, IL21-AS1 increased HIF1α-induced CD24 expression under hypoxic conditions. In parallel, we found that IL21-AS1 acted as a competing endogenous RNA (ceRNA) for miR-561-5p to regulate CD24 expression. Finally, IL21-AS1 increased CD24 expression in OC and facilitated OC progression. Our findings provide a molecular basis for the regulation of CD24, thus highlighting a potential strategy for targeted treatment of OC.

## Introduction

Epithelial ovarian cancer (OC), characterized by a high rate of relapse and poor prognosis, is the most lethal carcinoma of the female reproductive system [1], and these characteristics have prompted efforts towards identifying potential biomarkers and novel treatments for this disease. Long noncoding RNAs (lncRNAs) are a type of noncoding RNA with a typical length of more than 200 nucleotides [2]. Numerous lncRNAs whose expression is often aberrant in various cancers have been shown to be involved in tumour initiation, progression, and metastasis [3, 4]. LncRNAs have great potential as therapeutic targets in cancer. Therefore, it is important to elucidate the pathophysiological contributions of lncRNAs in OC.

Numerous studies have suggested that increased cluster of differentiation 24 (CD24) expression in tumours predicts poor prognosis [5, 6]. Recent studies have indicated that surface CD24 in tumours is a novel antiphagocytic protein that interacts with sialic-acid-binding Ig-like lectin 10 (Siglec10) on macrophages. Because of this intriguing function, CD24 is a novel target for cancer immunotherapy [7]. In preclinical animal models, monoclonal antibody (mAb)-mediated blockade of CD24 has been shown to be a promising therapeutic strategy for OC [7–9]. However, CD24 is expressed on and regulates many different types of normal cells [10, 11]. For example, it is normally expressed on cells of the immune system and the nervous system [12, 13]. Deficiency of CD24 on dendritic cells causes rapid homoeostatic proliferation of T cells when lymphopenia is induced, which leads to the death of immunocompetent mice [14]. Thus, direct usage of anti-CD24 mAbs may have unexpected side effects in patients. Therefore, it is necessary to further explore the molecular regulatory mechanisms of CD24, particularly in the tumour microenvironment (TME), the local biological environment of solid tumours [15].

TME is composed of cancer cells, vasculature, nearby stromal cells, and immune cells [15]. TAMs are the most predominant immune population of the TME, representing ~50% of hematopoietic cells [16, 17], and correlate with poor prognosis in human malignant tumours [18]. In OC, TAMs are the most abundant population of immune cells and organise an exceedingly immunosuppressive TME in OC via multiple mechanisms [19–22]. TAMs can express Siglec10, an inhibitory I-type lectin, which binds the don’t-eat-me signal CD24 and promotes immune evasion by inhibiting phagocytosis [7]. As the rapid and uncontrolled proliferation of tumours, large areas become deprived of oxygen and nutrients, which make hypoxia a typical microenvironment feature in nearly all solid tumours [23]. M2-like TAMs can be enriched in hypoxic tumour areas, and their population increases with tumour progression [24–26].

Here, we focus on IL21 antisense RNA 1 (IL21-AS1), a lncRNA that is transcribed from the antisense strand of the IL21 gene locus and has not been reported in tumours, was more highly expressed in OC samples than in normal ovarian tissue. We found that upregulation of IL21-AS1 inhibited the phagocytosis of TAMs and promoted OC tumorigenesis by enhancing CD24 expression in OC cells. IL21-AS1 deficiency reversed the immunosuppressive activity of TAMs and suppressed OC tumorigenesis by impairing CD24. We explored the possible mechanisms by which IL21-AS1 regulates CD24 under normoxic and hypoxic conditions. These results identify IL21-AS1 as a cancer biomarker regulating CD24-related phagocytosis inhibition and tumorigenesis, indicating its potential as a therapeutic target for OC.

## Materials and methods

### Patient samples

OC tissues (*n* = 43) and normal ovarian tissues (*n* = 40), stored in liquid nitrogen, were obtained from the Department of Biobank of Shanghai First Maternity and Infant Hospital. OC was staged by experienced pathologists at Shanghai First Maternity and Infant Hospital. The pathological diagnosis of all samples was clear. The clinical characteristics of the ovarian cancer patients documented in the supplementary Table S1.

### Bioinformatics analysis

In this research, the expression profile of 379 ovarian cancer was downloaded from TCGA (https://www.cancer.gov/ccg/access-data Accession date: 2020-10-2) and data was normalized by edger R package. Then the Pearson relation between IL21-AS1 and other genes was computed by Cor packge. The top 300 positively related and top 300 negatively related genes were enroled for Gene Ontology (GO) analysis to predict the function of IL21-AS1 in ovarian cancer. Further, the expression profiles of 379 ovarian cancer were quartered according to the expression level of IL21-AS1. Then, the top and bottom IL21-AS1 expression group were enroled for infiltrated immune cell analysis(http://bioinfo.life.hust.edu.cn/ImmuCellAI Accession date: 2020-11-7) [27, 28].

### Cell culture and treatments

OC cell line SKOV3 and ES2, human leukaemia monocytic cell line THP-1 were purchased from the Cell Bank of the Chinese Academy of Sciences (Shanghai, China), and OC cell line A2780 cells were a gift from Dr Zhang (Gynecology Hospital of Fudan University, Shanghai, China). SKOV3, A2780 and THP-1 cells were cultured in RPMI-1640 medium (Cat No. KGM31800N-500, KeyGEN, China). ES2 cells were cultured in DMEM (Cat No. KGM12800-500, KeyGEN, China). All cells were supplied with 10% foetal bovine serum (Cat No.10099141C, Gibco, USA) and 100 U/mL penicillin/streptomycin (Cat No. ST488S, Beyotime, China) in the culture medium and were cultured in a humidified 37°C incubator with 5% CO_2_.

### Macrophage generation

Macrophages used for all in vitro phagocytosis assays were differentiated from THP-1 cells. THP-1 cells were plated into six-well plates at a density of 3 × 10^6^ cells/well and treated with 100 ng/mL phorbol 12-myristate-13-acetate (PMA) (Cat No. P8139, Sigma‒Aldrich, USA) for 48 h. Then, human IL-4 (Cat No. 200-04, Peprotech, USA) and IL-10 (Cat No. 200-10, Peprotech, USA) were added at a concentration of 20 ng/mL for stimulation for 72 h.

### Cell transfection

For IL21-AS1 overexpression, the pLenti-CMV-IL21-AS1-GFP-Puro lentiviral vector and empty vector were obtained from PPL (Public Protein/Plasmid Library, China). For IL21-AS1 downregulation, the pPLK/GFP-Puro-IL21-AS1 lentiviral vector containing a targeting shRNA (target sequence GGTCCTCATAGTTCTTTCTGT) and a vector containing a scrambled shRNA were purchased from PPL. With reference to a protocol described previously [29], lentiviruses were generated by triple transfection of the above plasmids and helper plasmids into 293-T cells and were then purified from the cell culture medium. Cells were plated at 1 × 10^5^ cells/mL in 6-well plates and infected with lentivirus in the presence of 8 µg/mL polybrene (Cat No. C0351, Beyotime, China). Three days after transduction, GFP-positive polyclonal cells were screened with puromycin (Cat No. ST551, Beyotime, China). Then, GFP-positive sh-IL21-AS1 SKOV3 and OE-IL21-AS1 SKOV3/A2780/ES2 cell lines and the corresponding control cell lines were used in follow-up experiments. To overexpress IL21-AS1 and knock down CD24 or HIF1A, pLKO.1-NEO-CD24 (target sequence ACAACTGGAACTTCAAGTAAC), pLKO.1-NEO-HIF1A (target sequence CCAGTTATGATTGTGAAGTTA) and a negative control vector were synthesized by Tsingke (Beijing, China). OE-IL21-AS1 SKOV3/A2780/ES2 cells were infected with lentivirus containing CD24 shRNA, HIF1A shRNA or control shRNA. Positive polyclonal cells were screened with G418 (Cat No. G4024, Servicebio, China).

### Animals

All animal experiments were in accordance with the protocol approved by the Institutional Animal Care and Use Committee (IACUC) of Tongji University (Ethics Committee number: TJBG01723103). Female BALB/c nude mice (4–5 weeks old) were purchased from the SLAC Laboratory Animal (Shanghai, China). Mice were bred at the specific pathogen-free animal facilities of the Animal Research Center of Tongji University (Shanghai, China) in compliance with the Guide for the Care and Use of Laboratory Animals. In all experiments, mice were randomly divided into different groups.

For tumour growth experiments in vivo, 5 × 10^6^ target cells in 100 μL of PBS were injected subcutaneously into mice. Tumour growth was monitored by measuring the tumours at regular intervals using a calliper, and tumour volume was calculated using the following equation: volume = length × width^2^ × 0.52 mm^3^. The maximal tumour diameter was 1.5 cm. The nude mice were sacrificed, and tumour tissues were harvested, weighed, and formalin-fixed for subsequent immunohistochemical staining. Part of each tumour was stored in a −80°C freezer for follow-up experiments.

For survival analyses, deaths were recorded on the days on which the primary tumour burden reached 2 cm and/or the mouse was extremely emaciated, with weight loss exceeding 20% of the initial body weight.

### Flow cytometry

Single-cell suspensions of 5 × 10^6^ target cells with endogenous GFP fluorescence were implanted into 4–5-week-old female mice via intraperitoneal injection in 500 μL of PBS. After 3 weeks, cells were collected by peritoneal lavage with cold PBS. The cell suspensions were filtered through a 100-μm filter and centrifuged at 960 × *g* for 5 min at 4 °C. After lysed red blood cells with 1 ml Lysing Buffer (Cat No. A049201, Thermo Fisher Scientific, USA) for 5 minutes at room temperature, washed with cold PBS, 2 × 10^6^ cells were blocked with monoclonal antibody to CD16/32 (Cat No. 101302, BioLegend, USA) for 15 min at 4 °C. Then, cells were labelled with antibody panels (1:100) for 30 min at 4 °C and subsequently washed with FACS buffer. Flow cytometry was performed on BD FACS Calibur platforms, and 3 × 10^4^–5 × 10^4^ cells were analyzed for each group.

For the in vitro cell samples, 1.5 × 10^5^–3 × 10^5^ cells were collected from plates and washed with cold PBS. Then, cells were labelled with the antibody panels (1:100) for 30 min at 4 °C. Flow cytometry was performed on BD FACS Canto ΙΙ platforms, and 1 × 10^4^ cells were analyzed for each group.

### Phagocytosis assay

The in vitro phagocytosis assays were performed by coculturing target GFP-positive cells and macrophages at a ratio of 120,000 target cells to 60,000 macrophages for 1–2 h in ultralow-attachment 96-well plates (Cat No. 3473, Corning, USA) in serum-free RPMI-1640 medium in a humidified 5% CO_2_ incubator at 37 °C. After co-culture, reactions were centrifuged at 960 × *g* for 2 min at 4 °C, and then stained with an APC-labelled anti-CD45 antibody (Cat No. 982314, BioLegend, USA) to identify human macrophages. Cells were analyzed by flow cytometry on a BD FACSCanto ΙΙ instrument. Phagocytosis was quantified as the number of CD45^+^ and GFP^+^ macrophages as a percentage of the total number of CD45^+^ macrophages.

For the in vivo phagocytosis assay, cells were harvested by peritoneal lavage as described above. Cells were stained with anti-CD45-PerCP (Cat No. 103132, BioLegend, USA), anti-CD11b-APC (Cat No. 101212, BioLegend, USA), and anti-F4/80-PE (Cat No. 123110, BioLegend, USA) antibodies. Data were collected with a FACSCalibur flow cytometer (BD Biosciences). Phagocytosis was quantified as the percentage of CD11b^+^F4/80^+^ TAMs that were also GFP-positive. The gating strategy is shown in Fig. S2.

### CCK8 assay

Cell proliferation was measured by a Cell Counting Kit-8 (Cat No. BS350B, Biosharp, China). A total of 3000 target cells were seeded into 96-well plates (100 μL/well) and conventionally cultured for 24–96 h. After the cells were cultured for different periods, CCK8 solution (10 µL CCK8 reagent +90 μL medium) was added to the cells in each well for 2 h. The absorbance at 450 nm was measured to evaluate cell proliferation.

### EdU incorporation assay

In the in vitro EdU incorporation assay, cell proliferation was measured by an EdU Cell Proliferation Kit with Alexa Fluor 555 (Cat No. CX003, CellorLab, China) according to the manufacturer’s protocol.

In the in vivo EdU incorporation assays, mice were intraperitoneally injected with 200 µg of EdU reagent (Cat No. C0081S, Beyotime, China) in PBS. After 4 h, cells were collected by peritoneal lavage as described above. Cells were stained with an anti-CD45-PerCP antibody, and then the manufacturer’s instructions were followed for the remaining steps. Data were collected with a FACSCalibur flow cytometer and analyzed by FlowJo software (FlowJo™, 10.9). The gating strategy is shown in Fig. S2.

### Apoptosis assay

For the in vitro apoptosis assay, an Annexin V-APC/7-AAD Apoptosis Detection Kit (Cat No. KGA1026, KeyGEN, China) was used according to the manufacturer’s protocol.

For the in vivo apoptosis assay, cells were obtained by peritoneal irrigation and stained with an anti-CD45-PE-cy7 antibody (Cat No. 103114, BioLegend, USA). Then, a PE Annexin V Apoptosis Detection Kit (Cat No. 559763, BD, USA) was used in accordance with the instructions. The gating strategy is shown in Fig. S2.

### Western blotting (WB)

Total protein samples were extracted from OC cell lines and tissues using RIPA (Cat No. BL504A, Biosharp, China) lysis buffer supplemented with protease/phosphatase inhibitors (Cat No. BL612A, Biosharp, China). Proteins (20 μg/lane) were separated using 10–12% SDS‒PAGE and then transferred onto a PVDF membrane (Cat No. IPVH00010, Millipore, USA). The membrane was then blocked in 5% nonfat milk and incubated overnight with specific primary antibodies at 4 °C. The primary antibodies included anti-β-Actin (1:50,000; Cat No. AC026, ABclonal, Wuhan, China), anti-GAPDH (1:2000; Cat No. 60004-1-Ig, Proteintech, Wuhan, China), anti-CD24 (1:1000; Cat No. AP22227c, Abcepta, Suzhou, China), anti-CD47 (1:1000; Cat No. EM1701-37, HUABIO, Hangzhou, China), anti-HIF-1α (1:2000; Cat No. 79233, CST, USA), anti-p-STAT3 (1:1000; Cat No. 9145, CST, USA), anti-STAT3 (1:1000; Cat No. 4904, CST, USA), anti-Caspase 3/p17/p19 (1:1000; Cat No. 19677-I-AP, Proteintech, Wuhan, China) and anti-C-Myc (1:1000; Cat No. T55150, Abmart, Shanghai, China). After incubation with the corresponding secondary antibodies, bands were detected using ECL chemiluminescence substrate (Cat No. BL520A, Biosharp, China).

### Quantitative real-time PCR (qRT‒PCR)

Total RNA was extracted from tissues and cells using the TRIzol reagent kit (Cat No. 15596018, Invitrogen, USA) and converted into cDNA using the Hifair® Advance Fast 1st Strand cDNA Synthesis Kit (Cat No. 11149ES, Yeasen, China) or Mir-X miRNA First-Strand Synthesis Kit (Cat No. 638315, Takara, Japan) according to the manufacturer’s instructions. qRT-PCR was performed using a TB Green qPCR Premix Kit (Cat No. RR820A, Takara, Japan) on a qRT-PCR instrument (Applied Biosystems, USA). ACTB and U6 were used as controls for quantification of mRNA and miRNA levels, respectively. The primer sequences were as follows: IL21-AS1-F GAGTGTTGGGCTTTCTCCAA, IL21-AS1-R ACACCAGGGTCATAGGCAAC; CD24-F CTCCTACCCACGCAGATTTATTC, CD24-R AGAGTGAGACCACGAAGAGAC; CD47-F: AGAAGGTGAAACGATCATCGAGC, CD47-R: CTCATCCATACCACCGGATCT; PD-L1-F: TGGCATTTGCTGAACGCATTT, PD-L1-R: TGCAGCCAGGTCTAATTGTTTT; B2M-F: GAGGCTATCCAGCGTACTCCA, B2M-R: CGGCAGGCATACTCATCTTTT; miR-561-5p: ATCAAGGATCTTAAACTTTGCC, the 3′ primer for qRT‒PCR was the mRQ 3′ Primer supplied with the kit; ACTB-F CCATTGGCAATGAGCGGTTCC, ACTB-R CGGATGTCCACGTCACACTTCA. The agarose electrophoresis analysis and Sanger sequencing were used to determine the specificity of the response.

### Immunofluorescence (IF) staining

OC cell lines were cultured in confocal dishes (Cat No. BS-20-GJM, Biosharp, China) and then fixed with 4% paraformaldehyde in PBS for 15 min. The cells were permeabilized with 0.5% Triton X-100 in PBS for 10 min and then incubated in 5% BSA for 1 hr. The cells were then incubated overnight at 4 °C with anti-CD24 (1:100; Cat No. ab202073, Abcam, UK). The secondary antibody anti-rabbit IgG (H + L) Alexa Fluor 555 (Cat No. ab150074, Abcam, UK) was used at a 1:500 dilution for 1 h. Antifade mounting medium with DAPI (Cat No. C1002, Beyotime, China) was used for nuclear staining. Then, the cells were imaged on a Leica confocal microscope.

### Immunohistochemical (IHC) analysis

After deparaffinization in a xylene series and rehydration through a series of descending ethanol concentrations, tumour sections were heated in 0.01 M sodium citrate buffer (pH 6.0) for antigen retrieval and then treated in 3% H_2_O_2_ for 25 min to block endogenous peroxidase. Sections were blocked with 3% BSA for 30 min and then incubated with anti-Ki67 antibodies (1:300; Cat No. GB121141, Servicebio, China) overnight at 4 °C. On the next day, the sections were washed and incubated with secondary antibodies for 50 min at room temperature. Finally, the sections were stained with a DAB chromogen kit and counterstained with haematoxylin.

### Fluorescence in situ hybridization (FISH) and IF

An in situ hybridization kit was used (Cat No. GF002, Servicebio, China) according to the manufacturer’s instructions. In brief, following deparaffinization and rehydration, sections were subjected to digestion with proteinase K for 15 min at 37 °C. Then, the sections were hybridized to the FISH probe (500 nM) at 40 °C overnight, and finally, the signal probe hybridization solution was added for 45 min at 40 °C. After washing with SSC buffer, the sections were blocked with 5% BSA at room temperature for 30 min. The subsequent IF protocols were performed as previously described. The primary antibodies included anti-CD24 (1:100; Cat No. ab202073, Abcam, UK) and anti-HIF-1α (1:200; Cat No. 79233, CST, USA), and the corresponding secondary antibodies were Cy5-conjugated goat anti-rabbit IgG (H + L) (1:400; Cat No. GB27303, Servicebio, China) and Cy5-conjugated goat anti-mouse IgG (H + L) (1:400; Cat No. GB27301, Servicebio, China), respectively. The FISH probe for IL21-AS1 was designed and synthesized by Servicebio, and the probe sequences were 5′-CCATTCCTCTTCAGACTTCTACCCCT-3′ and 5′-AGTTTTAGACCTGGCACAGTTTTCCC-3′.

### Dual luciferase reporter assay

The wild-type (WT) or mutated (Mut) fragment of IL21-AS1 and the CD24 3′ untranslated region (3′-UTR) were inserted downstream of the pmirGLO vector (PPL, China). The human miR-561-5p mimic (miR-561-5p-mimic) and negative control mimic (NC-mimic) were also obtained from PPL. A2780 and ES2 cells were cotransfected with the recombinant vectors and miR-561-5p-mimic or NC-mimic by using Lipo2000 (Cat No. 11668019, Invitrogen, USA). Twenty-four hours later, the firefly luciferase and Renilla luciferase activity in each transfection group were measured by using the Dual Luciferase Reporter Gene Assay Kit (Cat No. E1910, Promega, USA).

### Statistical analysis

All experiments were conducted by using 5–7 mice or repeated three independent times with cells. All results are shown as mean ± SD or mean ± SEM. Student’s t test, one-way ANOVA and two-way ANOVA were used to determine the statistical significance of differences between groups. Kaplan–Meier survival analysis was used to compare mouse survival in GraphPad Prism software V8.0. *P* < 0.05 was considered to indicate a statistically significant difference.

## Results

### IL21-AS1 is upregulated in OC tissues and probably affects macrophage-mediated phagocytosis

**Fig. 1:**
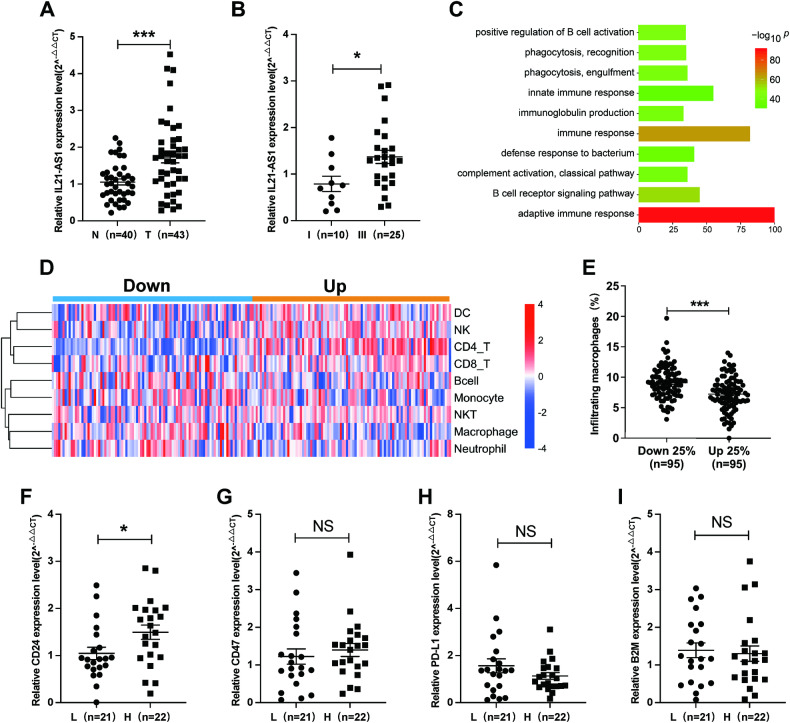
IL21-AS1 is upregulated in OC tissues and probably affects macrophage-mediated phagocytosis. **A** Expression levels of IL21-AS1 in normal (N, *n* = 40) and OC (T, *n* = 43) tissues as determined by RT‒qPCR. **B** RT-qPCR for IL21-AS1 expression in patients with stage I (*n* = 10) or III (*n* = 25) disease classified by FIGO stage. **C** GO functional enrichment analysis based on IL21-AS1 expression in OC tissues from the TCGA database. **D** Analysis of tumour-infiltrating immune cells in OC patients in a TCGA cohort based on the IL21-AS1 level. **E** Macrophage infiltration in OC patients in a TCGA cohort according to the IL21-AS1expression level (*n* = 95 vs. 95). Relative mRNA levels of CD24 (**F**), CD47 (**G**), PD-L1 (**H**), B2M (**I**) between IL21-AS1^High^ OC patients (**H**, *n* = 22) and IL21-AS1^Low^ OC patients (L, *n* = 21) as determined by RT-qPCR. RT-qPCR data are normalized to ACTB (**A**, **B**, **F**–**I**). The data are expressed as the mean ± SEM. Statistical significance was accessed by unpaired t test *P < 0.05, **P < 0.01, ***P < 0.001, NS not significant.

To evaluate IL21-AS1 expression in OC tissues, we examined the expression of IL21-AS1 in OC and normal ovarian tissues (43 vs. 40) and found that IL21-AS1 expression was dramatically increased in OC tissues (Fig. 1A). In addition, we confirmed that IL21-AS1 expression was significantly upregulated in patients with stage III disease compared with patients with stage I disease (25 vs. 10) (Fig. 1B). To identify the potential biological characteristics of IL21-AS1, the Cor R package was used to analyse the correlations between IL21-AS1 and other genes in the OC cohort in the TCGA database. Gene Ontology (GO) enrichment analysis revealed that IL21-AS1 was highly involved in the immune response, mainly in regulating the innate immune response (Fig. 1C). In order to further exploring the type of immune cells that IL21-AS1 mainly influenced, ImmuneCellAI was used to further identify the infiltrated immune cells in each OC tissue. Then, data from the TCGA OC cohort were used for analysis of immune cell infiltration. The cancer tissues with IL21-AS1 expression in the top quartile were assigned to the upregulated group, and the cancer tissues with IL21-AS1 expression in the bottom quartile were assigned to the downregulated group. As shown in Fig. 1D, the infiltration of CD4^+^ T cells, CD8^+^ T cells, NK cell, NK T cell and dendritic cell was decreased in the downregulated group. However, macrophage and neutrophil infiltration was increased in OC tissues with lower IL21-AS1 expression, and the macrophage infiltration was elevated greater than neutrophil infiltration (Fig. 1D, E and Table S2). As mentioned above, IL21-AS1 mainly regulates the innate immune response. Among the involved biological processes, phagocytosis is the key function of macrophages in the TME. Cancer cells can evade clearance by macrophages through overexpression of antiphagocytic surface proteins including CD47 [30], programmed cell death ligand 1 (PD-L1) [31], the beta-2 microglobulin subunit of the major histocompatibility class I complex (B2M) [32], and CD24 [7]. OC samples were grouped to IL21-AS1^Low^ and IL21-AS1^High^ cases based on IL21-AS1 median cut-off, and we compared the expression levels of those four phagocytosis checkpoints by using qRT-PCR (Fig. 1F–I). IL21-AS1^High^ samples exhibited a significant higher level of CD24 mRNA (Fig. 1F). These results demonstrated that IL21-AS1 expression was upregulated in OC tissues and probably involved in macrophage-mediated phagocytosis.

### IL21-AS1 inhibits macrophage-mediated phagocytosis

**Fig. 2:**
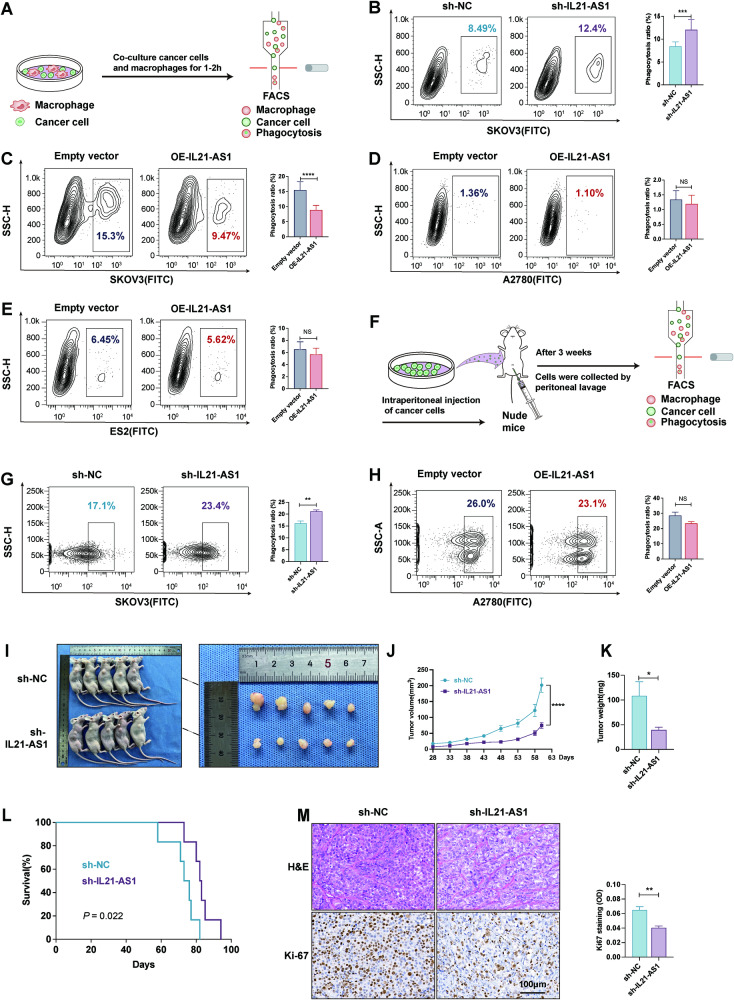
IL21-AS1 inhibits macrophage-mediated phagocytosis, and its deficiency suppresses tumour growth in the OC cell line SKOV3. **A** Schematic protocol for the in vitro phagocytosis assay. **B** Flow cytometry-based measurement of phagocytosis of sh-IL21-AS1 SKOV3 cells and sh-NC SKOV3 cells by cocultured macrophages. Left, representative images. Right, statistical analysis. *n* = 3, biological replicates. **C**–**E** Phagocytosis of OE-IL21-AS1 SKOV3 (**C**), A2780 (**D**), ES2 (**E**) cells and control cells by cocultured macrophages. Left, representative images. Right, statistical analysis. *n* = 3, biological replicates. **F** Schematic protocol for in vivo macrophage-mediated phagocytosis of OC cells. In vivo phagocytosis of sh-IL21-AS1 SKOV3 cells (**G**), OE-IL21-AS1 A2780 cells (**H**) and control cells by macrophages (*n* = 6 in each group). Left, representative images. Right, statistical analysis. Photographs (**I**), volumes (**J**), and weights (**K**) of tumours in mice injected subcutaneously with sh-IL21-AS1 SKOV3 cells or sh-NC cells (*n* = 5 in each group). **L** Survival analysis of mice injected subcutaneously with sh-IL21-AS1 SKOV3 cells or sh-NC cells (*n* = 7 in each group). **M** Proliferation of engrafted tumours in (**I**). Upper, H&E staining; Lower, immunohistochemical staining of Ki67; Right, quantitative analysis of Ki67 expression. Scale bar, 100 μm. All results are shown as mean ± SD (**B**–**E**) or mean ± SEM (**G**, **H**, **J**, **K**, **M**). Statistical significance was accessed by Student’s t test (**B**–**E**, **G**, **H**, **K**, **M**), two-way ANOVA (**J**), and log-rank test (**L**). *P < 0.05, **P < 0.01, ***P < 0.001, ****P < 0.0001, NS not significant.

As mentioned above, we hypothesized that IL21-AS1 could affect phagocytosis by macrophages. We engineered GFP-positive polyclonal sublines with silencing or overexpression of IL21-AS1 in OC cell lines (Fig. S1). Those sublines and the corresponding controls were cocultured with THP-1 cell-derived macrophages for 1–2 h. Then, macrophage-mediated clearance of tumour cells was detected by flow cytometry (Fig. 2A). Downregulation of IL21-AS1 in SKOV3 cells was sufficient to potentiate macrophage phagocytosis (Fig. 2B). We overexpressed IL21-AS1 in three different OC cell lines, SKOV3, A2780 and ES2. After coculturing these three OE-IL21-AS1 sublines with macrophages, we observed significantly decreased macrophage phagocytosis only in the SKOV3 polyclonal cultures (Fig. 2C). Interestingly, overexpression of IL21-AS1 in A2780 and ES2 cells did not change tumour cell evasion of phagocytic clearance (Fig. 2D, E). To further investigate the role of IL21-AS1 in regulating macrophage-mediated phagocytosis, OE-IL21-AS1 A2780 cells, sh-IL21-AS1 SKOV3 cells and the corresponding control cells (5 × 10^6^) were injected intraperitoneally into female nude mice. After three weeks, cells were collected by peritoneal lavage (Fig. 2F). Loss of IL21-AS1 in SKOV3 cells significantly increased their phagocytic clearance by macrophages in mice (Fig. 2G). Consistent with the above results, overexpression of IL21-AS1 in A2780 cells had no effect on macrophage-mediated phagocytosis (Fig. 2H).

To observe the effects of IL21-AS1 on tumour growth more intuitively, we generated xenografts by subcutaneously injecting sh-NC and sh-IL21-AS1 SKOV3 cells (5 × 10^6^) into nude mice (*n* = 5 mice per group) as described in the methods. After ~8 weeks, the xenograft tumour volume and weight were significantly lower in the sh-IL21-AS1 group than in the control (sh-NC) group (Fig. 2I–K). In addition, this growth difference ultimately resulted in a significant survival advantage for mice engrafted with sh-IL21-AS1 cell-derived tumours (*n* = 7 mice per group) (Fig. 2L). We next sought to determine whether any cause in addition to the increase in phagocytosis induced by downregulation of IL21-AS1 led to the differences between the two groups. IHC analysis of xenograft tumours showed that silencing of IL21-AS1 caused a reduction in Ki67 expression, which suggested a decrease in cell proliferation (Fig. 2M). These data indicate that IL21-AS1 can inhibit the phagocytosis of SKOV3 cells by macrophages and can also promote SKOV3 cell proliferation. However, IL21-AS1 does not appear to have this effect on A2780 and ES2 cells.

### IL21-AS1 promotes OC cell proliferation and apoptosis inhibition

**Fig. 3:**
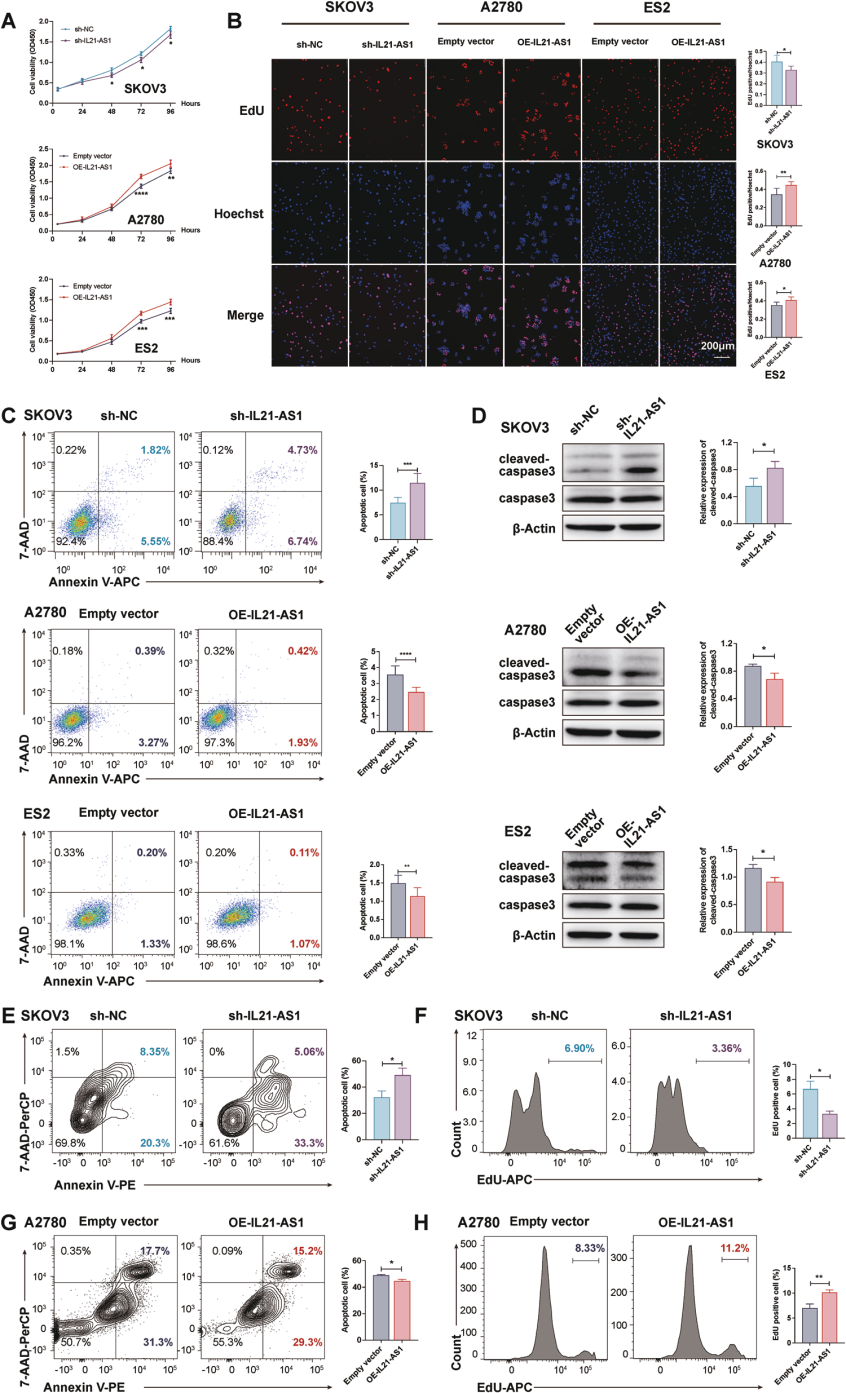
IL21-AS1 promotes OC cell proliferation and inhibits apoptosis. A CCK8 assay (**A**), an EdU incorporation assay (**B**) and an apoptosis assay (**C**) were performed in SKOV3, ES2 and A2780 cells with altered expression of IL21-AS1 in vitro. Left, representative images. Right, statistical analysis. Scale bar, 200 μm. *n* = 3, biological replicates. **D** Cleaved caspase3 protein level in cells in (**C**). Left, representative images. Right, statistical analysis. *n* = 3, biological replicates. **E**–**H**, sh-IL21-AS1 SKOV3 and OE-IL21-AS1 A2780 cells and the corresponding control cells were intraperitoneally injected into nude mice, and tumours were allowed to grow for 3 weeks. Cells were collected by peritoneal lavage. Flow cytometry-based analysis of apoptotic cells (**E**, **G**) and an EdU incorporation assay (**F**, **H**) were performed (*n* = 6 or 7 in each group). Left, representative images. Right, statistical analysis. All results are shown as mean ± SD (**A**–**D**) or mean ± SEM (**E**–**H**). Statistical significance was accessed by two-way ANOVA (**A**), and Student’s t test (**B**–**H**). *P < 0.05, **P < 0.01, ***P < 0.001, ****P < 0.0001.

Considering the expression of Ki-67 observed by IHC staining in the two groups of xenograft tumours, we further investigated the biological effect of IL21-AS1 on OC cells. In vitro, cancer cell proliferation and apoptosis were assessed by a CCK8 assay, an EdU incorporation assay and Annexin V/7-AAD staining. Knockdown of IL21-AS1 in SKOV3 cells inhibited proliferation and increased apoptosis. In contrast, overexpression of IL21-AS1 in A2780 and ES2 cells increased proliferation and resulted in a significant decrease in apoptosis (Fig. 3A, B, C). The cleaved caspase-3 protein level, as measured by WB, markedly increased or decreased with knockdown or overexpression of IL21-AS1, respectively, consistent with the apoptosis rates in the groups (Fig. 3D). To examine the role of IL21-AS1 in regulating OC cell proliferation and apoptosis inhibition in vivo, sh-IL21-AS1 SKOV3 cells, OE-IL21-AS1 A2780 cells and the corresponding control cells were injected intraperitoneally into nude mice (*n* = 6 or 7 mice per group). After three weeks, OC cells were collected, stained, and analyzed by flow cytometry. As expected, a significantly lower percentage of EdU-positive cells and a higher number of apoptotic cells were detected among sh-IL21-AS1 cells in vivo (Fig. 3E, F). Consistent data were obtained for OE-IL21-AS1 A2780 cells (Fig. 3G, H). These results provided additional supporting evidence to confirm the role of IL21-AS1 in tumorigenesis.

### IL21-AS1 regulates CD24 expression

**Fig. 4:**
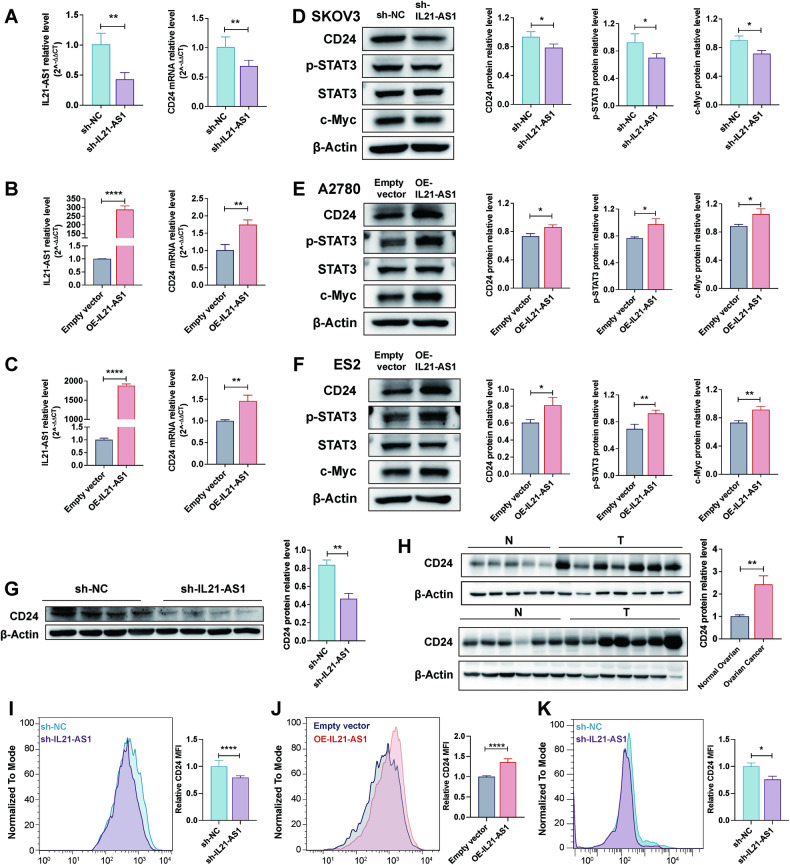
IL21-AS1 promotes CD24 expression. CD24 mRNA expression levels in SKOV3 (**A**), A2780 (**B**) and ES2 (**C**) cells with altered expression of IL21-AS1. *n* = 3, biological replicates. CD24, p-STAT3 and c-Myc protein levels in SKOV3 (**D**), A2780 (**E**) and ES2 (**F**) cells with altered IL21-AS1 levels. Left, representative images. Right, statistical analysis. *n* = 3, biological replicates. **G** CD24 protein expression level in xenograft tumours formed from SKOV3 cells with or without IL21-AS1 knockdown (*n* = 4 in each group). Left, representative images. Right, statistical analysis. **H** CD24 protein expression levels in normal (N, *n* = 11) and OC (T, *n* = 14) tissues. Left, representative images. Right, statistical analysis. Flow cytometry-based measurement of CD24 expression in sh-IL21-AS1 SKOV3 cells (**I**) and OE-IL21-AS1 SKOV3 cells (**J**). Left, representative images. Right, statistical analysis. *n* = 3, biological replicates. **K** sh-IL21-AS1 SKOV3 and control cells were intraperitoneally injected into nude mice, and tumours were allowed to grow for 3 weeks. Cells were collected by peritoneal lavage. Relative CD24 expression levels in two groups were determined by flow cytometry (*n* = 6 in each group). RT-qPCR data are normalized to ACTB (**A–C**). All results are shown as mean ± SD (**A**–**F**) or mean ± SEM (**G**–**K**). Statistical significance was accessed by Student’s t test. *P < 0.05, **P < 0.01, ***P < 0.001, ****P < 0.0001.

AS shown in Fig. 1F, a higher level of IL21-AS1 was related to a significant higher level of CD24 mRNA in OC tissues. Notably, the presence of CD24 impairs phagocytosis of OC cells by macrophages [7]. Then, we measured the CD24 mRNA and protein expression levels after knocking down or overexpressing IL21-AS1 in OC cells. As shown in Fig. 4A, the CD24 mRNA level was obviously reduced in sh-IL21-AS1 SKOV3 cells. Consistent with this finding, overexpression of IL21-AS1 in A2780 and ES2 cells resulted in a significant increase in the CD24 mRNA level (Fig. 4B, C). We then found that downregulation or overexpression of IL21-AS1 decreased or increased, respectively, the protein levels of CD24 and its downstream effectors, p-STAT3 and c-Myc [33, 34] (Fig. 4D–F). Western blot analysis also showed that the CD24 protein level was markedly decreased in sh-IL21-AS1 xenograft tumours (Fig. 4G). In addition, we measured the expression of CD24 in OC and normal ovarian tissues and found that CD24 protein level was dramatically increased in OC tissues (Fig. 4H).

Previous studies have shown that the intracellular localization of CD24 probably impacts tumour phenotypes [35]. Here, immunofluorescence staining revealed that CD24 was localized in the cytoplasm in SKOV3, A2780 and ES2 cells (Fig. S3A). FACS analysis showed that few A2780 and ES2 cells were surface CD24 positive, and almost all SKOV3 cells were surface CD24 positive (Fig. S3B). Further FACS analysis confirmed that knocking down or overexpressing IL21-AS1 in SKOV3 cells markedly decreased or increased the CD24 median fluorescence intensity (MFI), respectively, in vitro (Fig. 4I, J). Moreover, we determined the CD24 MFI in OC cells implanted in the mouse abdominal cavity. Compared with control cells, sh-IL21-AS1 SKOV3 cells had a lower cell surface CD24 signal in vivo (*n* = 6 mice per group) (Fig. 4K).

### IL21-AS1 upregulates CD24 expression through HIF-1α under hypoxic conditions

**Fig. 5:**
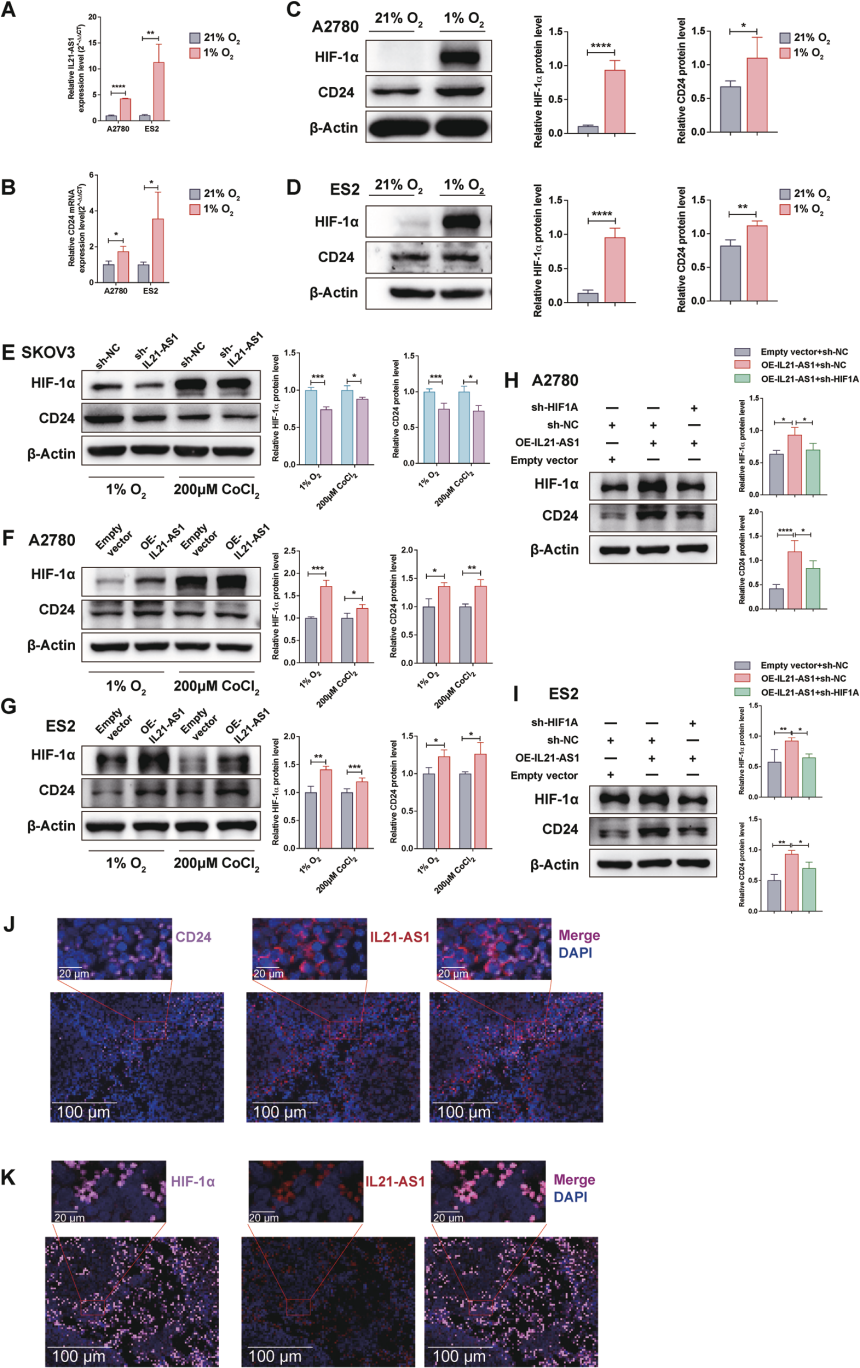
IL21-AS1 upregulates CD24 expression through HIF-1α under hypoxic conditions. **A** Relative levels of IL21-AS1 in A2780 and ES2 cells cultured under hypoxic conditions (1% O_2_ for 24 h). *n* = 3, biological replicates. **B** Relative CD24 mRNA levels in A2780 and ES2 cells under hypoxic conditions. *n* = 3, biological replicates. HIF-1α and CD24 protein levels in A2780 (**C**) and ES2 (**D**) cells under hypoxic conditions. Left, representative images. Right, statistical analysis. *n* = 3, biological replicates. HIF-1α and CD24 protein levels in SKOV3 (**E**), A2780 (**F**) and ES2 (**G**) cells with altered IL21-AS1 expression after exposure to 200 μM CoCl_2_ or culture under hypoxic conditions for 24 h. Left, representative images. Right, statistical analysis. *n* = 3, biological replicates. Relative protein levels of HIF-1α and CD24 in A2780 (**H**) /ES2 (**I**) cells with IL21-AS1 overexpression and HIF1A silencing. Left, representative images. Right, statistical analysis. *n* = 3, biological replicates. **J** FISH and IF assays revealed the overlapping localization of IL21-AS1 and CD24 in low-oxygen regions of xenograft tumours. Scale bar, 100 µm & 20 µm. **K** FISH and IF assays revealed the overlapping localization of IL21-AS1 and HIF-1α in low-oxygen regions of xenograft tumours. Scale bar, 100 µm & 20 µm. RT-qPCR data are normalized to ACTB (**A**, **B**). All results are shown as mean ± SD. Statistical significance was accessed by Student’s t test (**A**–**G**), and one-way ANOVA (**H**, **I**). *P < 0.05, **P < 0.01, ***P < 0.001, ****P < 0.0001.

Hypoxia is a typical microenvironmental feature in nearly all solid tumours. Thus, we exposed A2780 and ES2 cells to 1% O_2_ for 24 h. The qRT-PCR results showed that the IL21-AS1 and CD24 mRNA levels were increased in those two cell lines (Fig. 5A, B). Furthermore, WB revealed that the CD24 and HIF-1α protein levels were increased (Fig. 5C, D). However, CD24 has been identified as a transcriptional target of HIF-1a in hypoxia [36]. To determine the contributions of IL21-AS1 and HIF-1α to hypoxia-mediated induction of CD24 expression, three different OC cell lines transfected with sh-IL21-AS1 or OE-IL21-AS1 plasmids were cultured under hypoxic conditions or exposed to 200 μM CoCl_2_. IL21-AS1 overexpression dramatically increased the HIF-1α and CD24 protein levels (Fig. 5F, G). Consistent findings were observed in sh-IL21-AS1 SKOV3 cells (Fig. 5E). Then, OE-IL21-AS1 A2780 and ES2 cells were transfected with sh-HIF1A or control plasmids and cultured under hypoxic conditions. As expected, HIF1A downregulation reversed the IL21-AS1-induced increase in not only the HIF-1α protein level but also the CD24 protein level (Fig. 5H, I). Based on the above results, IL21-AS1 is a hypoxia-responsive lncRNA and that an IL21-AS1–HIF-1α–CD24 axis is likely activated under hypoxic conditions. To provide additional supporting evidence, IL21-AS1 FISH and CD24 or HIF-1α immunofluorescence staining were conducted with OE-IL21-AS1 A2780 xenograft tumour sections. The results revealed the colocalization of IL21-AS1 with CD24 and HIF-1α in the cytoplasm, and the signals were specifically increases in hypoxic tumour regions (Fig. 5J–K).

### IL21-AS1 regulates CD24 expression by sponging miR-561-5p

**Fig. 6:**
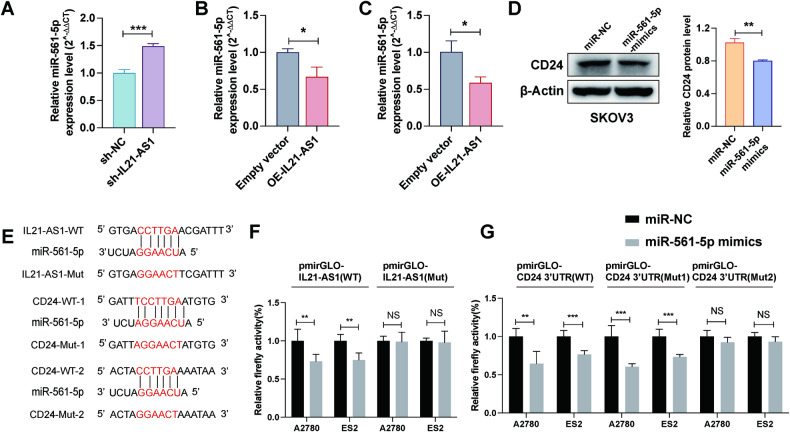
IL21-AS1 regulates CD24 expression by sponging miR-561-5p. Relative miR-561-5p expression levels in SKOV3 (**A**), A2780 (**B**), and ES2 (**C**) cells with altered expression of IL21-AS1. *n* = 3, biological replicates. **D** CD24 protein levels in SKOV3 cells with or without forced expression of miR-561-5p. Left, representative images. Right, statistical analysis. *n* = 3, biological replicates. **E** Complementarity between the miR-561-5p seed sequence and IL21-AS1 and the CD24 3′-UTR was identified by using the TargetScan and starBase online databases. Relative luciferase activity in A2780 cells and ES2 cells transfected with reporter plasmids containing the wild-type (WT) or mutant (Mut) miR-561-5p binding sites in IL21-AS1 (**F**) and the CD24 3′-UTR (**G**). *n* = 3, biological replicates. RT-qPCR data are normalized to U6 (**A**–**C**). All results are shown as mean ± SD. Statistical significance was accessed by Student’s t test. *P < 0.05, **P < 0.01, ***P < 0.001, NS not significant.

With two publicly available bioinformatics tools, TargetScan [37] (https://www.targetscan.org/vert_80/ Accession date:2022-11-07) and starBase [38, 39] (http://starbase.sysu.edu.cn/mirLncRNA.php Accession date:2022-11-07), we found that both IL21-AS1 and the 3′-UTR of CD24 contain the putative miR-561-5p binding site (Fig. 6E). As the qRT‒PCR data showed, miR-561-5p expression was negatively correlated with IL21-AS1 expression. Knockdown of IL21-AS1 resulted in upregulation of miR-561-5p (Fig. 6A). In contrast, overexpression of IL21-AS1 led to downregulation of miR-561-5p (Fig. 6B, C). Additionally, overexpression of miR-561-5p in SKOV3 cells dramatically decreased the CD24 protein level (Fig. 6D). Then, IL21-AS1 and the CD24 3’-UTR containing the miR-561-5p binding site were inserted into a luciferase reporter plasmid. Then, the resulting luciferase reporter plasmids were cotransfected with miR-561-5p-mimic or NC-mimic into A2780 and ES2 cells. Overexpression of miR-561-5p markedly attenuated the luciferase activity of the reporters containing the WT binding site of IL21-AS1 or the CD24 3′-UTR. Conversely, mutation of the target sequence blocked the inhibitory effect of miR-561-5p (we mutated two possible binding sites in the CD24 3′-UTR, and the Mut1 reporter was constructed similarly to the WT reporter) (Fig. 6F, G). These results suggested that IL21-AS1 could compete with CD24 for binding to miR-561-5p, thereby regulating the expression of CD24.

### Downregulation of CD24 reverses the IL21-AS1-induced effects on phagocytosis, cell proliferation and apoptosis inhibition

**Fig. 7:**
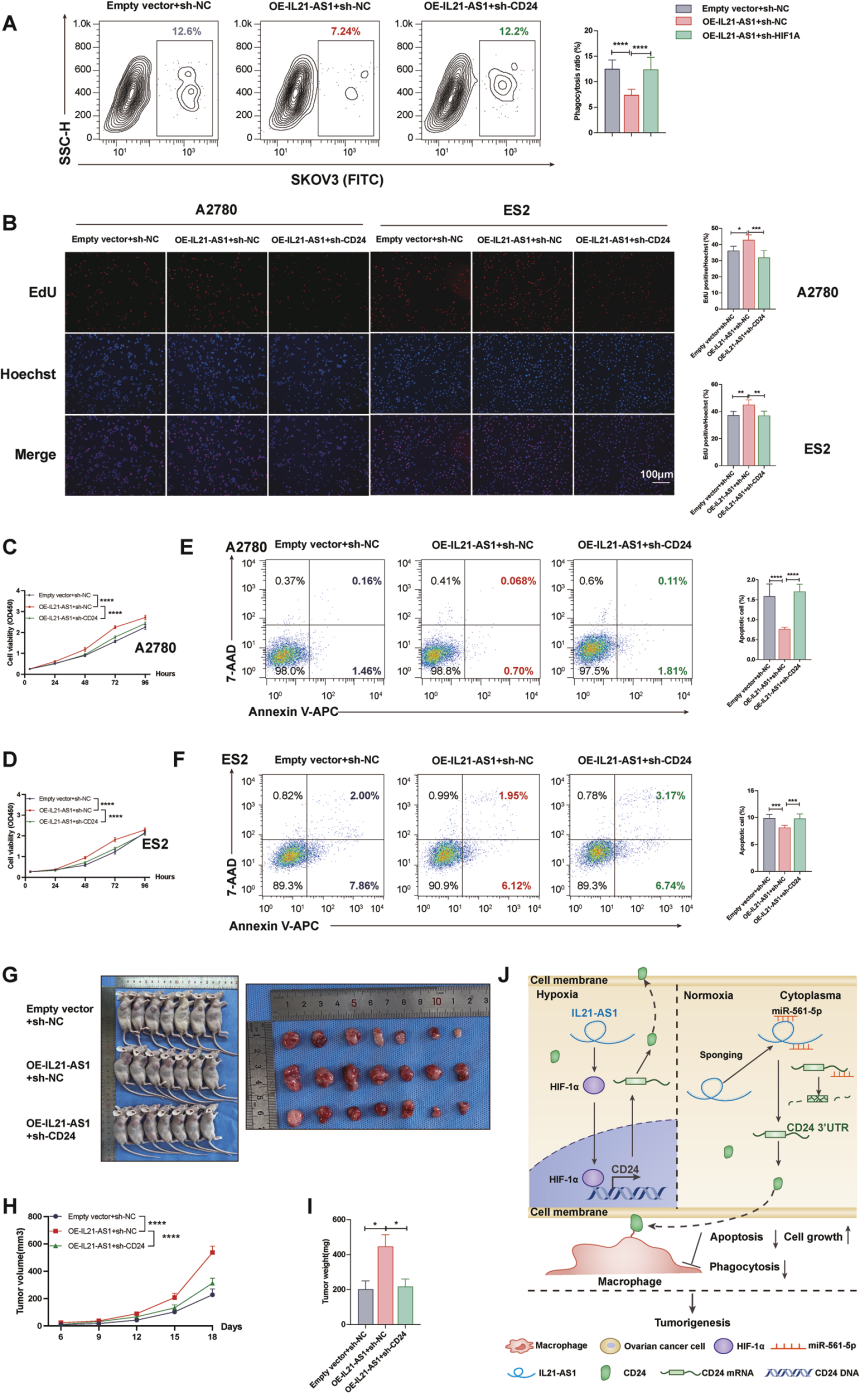
Downregulation of CD24 reversed the IL21-AS1-induced effects on phagocytosis, proliferation, and apoptosis inhibition. **A** Flow cytometry-based measurement of macrophage-mediated phagocytosis of SKOV3 cells with IL21-AS1 overexpression and CD24 silencing. Left, representative images. Right, statistical analysis. *n* = 3, biological replicates. EdU incorporation assay (**B**), CCK8 assay (**C**, **D**), and apoptosis assay (**E**, **F**) in A2780/ES2 cells with IL21-AS1 overexpression and CD24 silencing. Left, representative images. Right, statistical analysis. Scale bar, 100 μm. *n* = 3, biological replicates. **G–I** A2780 cells with IL21-AS1 overexpression and CD24 silencing, and the corresponding control cells were subcutaneously injected into nude mice (*n* = 7 in each group). Photographs (**G**), volumes (**H**), and weights (**I**) of tumours in three groups. **J** A proposed model of IL21-AS1 in promoting OC immune escape and tumorigenesis through up-regulating the expression of CD24. IL21-AS1 acts as a ceRNA to sponge miR-561-5p to regulate the expression of CD24 in the cytosol of cells. IL21-AS1 also up-regulates HIF1α-induced CD24 expression under hypoxic conditions. IL21-AS1-regulated CD24 protects OC cells from macrophage-mediated phagocytosis and promote ovarian tumorigenesis. All results are shown as mean ± SD (**A**–**F**) or mean ± SEM (**H**–**I**). Statistical significance was accessed by one-way ANOVA (**A**, **B**, **E**, **F**), and two-way ANOVA (**C**, **D**, **H**). *P < 0.05, **P < 0.01, ***P < 0.001.

To further confirm the regulatory effect of IL21-AS1 on CD24, a series of experiments were conducted after overexpressing IL21-AS1 in SKOV3, A2780 and ES2 cells and then knocking down CD24. CD24 knockdown in IL21-AS1-overexpressing SKOV3 cells restored their macrophage-mediated phagocytosis (Fig. 7A). Moreover, CD24 downregulation in A2780 or ES2 cells abrogated the effects of IL21-AS1 on proliferation and apoptosis inhibition (Fig. 7B–F). Finally, we conducted in vivo rescue experiments and found that silencing CD24 in IL21-AS1-overexpressing A2780 cells attenuated the IL21-AS1-mediated increases in tumour volume, weight, and growth rate (*n* = 7 mice per group in each assay) (Fig. 7G–I), consistent with the in vitro results.

## Discussion

Healthy cells display ‘don’t eat me’ signals, which bind to cognate inhibitory receptors, to prevent self-elimination [40], and malignant cells depend even more on similar mechanisms to evade immune-mediated eradication [41]. To date, antiphagocytic surface proteins identified on cancer cells include CD47 [30], programmed cell death ligand 1 (PD-L1) [31], beta-2 macroglobulin subunit of the major histocompatibility class I complex (B2M) [32]and CD24 [7]. With reference to these studies concerning phagocytosis checkpoints and considering the previously mentioned characteristics of TAMs within the OC TME, we differentiated macrophages with M2-polarizing cytokine IL-4 and IL-10 in this study. Our data showed that impaired the IL21-AS1 expression in SKOV3 cells enabled TAMs to attack cancer cells in vivo and in vitro. Consistent results were obtained upon IL21-AS1 overexpression in SKOV3 cells. As shown in earlier studies, high expression of CD47 and CD24 in OC tissues is correlated with poor clinical outcomes [42, 43]. Notably, in preclinical research related to OC, suppressing the expression of CD47 and CD24 or blocking them with anti-CD47 and anti-CD24 mAbs was found to enhance macrophage-mediated phagocytosis, thus resulting in a significant reduction in the growth of xenograft tumours [7, 44]. LncRNAs exert biological effects by regulating target genes. The significantly higher expression of CD24 mRNA in IL21-AS1^High^ OC tissues and the results of follow-up experiments suggested that IL21-AS1 regulated the expression of CD24 but not CD47 (Fig. S4). The levels of p-STAT3 and c-Myc, downstream effector proteins of CD24, were evaluated upon modulation of CD24 protein expression, and the corresponding changes in their expression reinforced the evidence that CD24 is regulated by IL21-AS1.

As shown in our data, although overexpression of IL21-AS1 in A2780 and ES2 cells increased the CD24 protein level, it did not alter macrophage-mediated clearance of these cancer cells, similar to the findings in the SKOV3 sublines tested. Phagocytosis of tumour cells is regulated through receptor–ligand interactions at the cell–cell interface [41, 45]. Although CD24 is generally known to be a glycosylphosphatidylinositol-anchored surface protein, cytoplasmic expression of CD24 was reported in previous studies related to OC [43, 46]. IF and flow cytometric analyses showed almost no CD24 expression on the A2780 and ES2 cell surface. The absence of the surface protein CD24 is a possible reason that IL21-AS1 did not inhibit the phagocytosis of A2780 and ES2 cells. In this study, we found that IL21-AS1 could promote proliferation and inhibit apoptosis in the A2780 and ES2 cell lines, which indirectly suggested a specific role of cytoplasmic CD24 in OC progression. Consistent with this finding, researchers have shown that the cytoplasmic accumulation of CD24 may increase prostate cancer cell proliferation by functional inactivation of p53 [47]. Our data indicated that the size of the surface CD24-positive population varied among different OC cell lines. Previous research showed that among 20 clones formed by primary human OC cells, 0.4**–**69.2% of the cells exhibited surface CD24 expression [48]. Hence, tumours from some patients are at least partially negative for surface CD24. This observation opposes the premise that anti-CD24 antibody-based targeted therapies are effective as monotherapies. Interestingly, IL21-AS1 could regulate global CD24 expression. This observation highlights a distinct advantage of IL21-AS1 as a therapeutic target in OC.

Hypoxia is a hallmark of solid tumours, and the HIF pathway is the most widely recognized pathway enabling cancer progression in the hypoxic TME [23]. HIF-1α is associated with invasion, metastasis, and platinum resistance in OC [49, 50] and is related to worse survival outcomes [51]. In this study, we observed that IL21-AS1 was upregulated under hypoxic conditions and that IL21-AS1 overexpression dramatically increased the HIF-1α and CD24 protein levels. Considering that CD24 was reported to be transcriptionally activated by HIF-1α [36], our studies with sh-HIF1A, revealed the existence of an IL21-AS1–HIF-1α–CD24 axis in OC cells. In addition, the colocalization of IL21-AS1, HIF-1α, and CD24 in necrotic tumour regions further confirmed this hypothesis. The efficacy of targeting CD24 in OC has been assessed in several preclinical trials [7, 9, 52]. The anti-CD24 mAbs evaluated in these trials target the sequence in human CD24 but not the murine CD24 homologue. The application of anti-CD24 mAbs in patients may have unexpected side effects, given the expression of CD24 on many normal cell types. LncRNAs are often characterized by low expression levels [53]. Using of small interfering RNAs (siRNAs) and antisense oligos (ASOs) to silence and target specific RNAs presents great potential for RNA therapy [54, 55]. Small molecule drugs based on ribonuclease-targeting chimeras (RIBOTAC) technology also makes it possible to degradation of target RNAs [56, 57]. Drugs specifically targeting IL21-AS1 to interfere with the expression of IL21-AS1 probably lead to specific downregulation of CD24 in tumours. It may provide a new avenue for the therapy of OC in clinic, which needs further investigation.

Chronic hypoxia and diffusion-restricted hypoxia cause the necrotic death of tumour cells within a 180-μm periphery of blood vessels; thus, solid tumours often present with both hypoxic and nonhypoxic regions [58, 59]. Moreover, there should be other pathways through which IL21-AS1 regulates CD24 expression. Based on their subcellular distribution, lncRNAs exert biological effects by regulating target gene expression at different levels. LncRNAs in the cytoplasm modulate mRNA stability, protein translation and posttranslational modification [60] via mechanisms such as acting as competing endogenous RNAs (ceRNAs), which function as miRNA sponges by competing for miRNA binding [53, 61, 62]. Here, FISH staining in xenograft tumour sections showed that IL21-AS1 was localized mainly in the cytoplasm of A2780 cells. Through bioinformatics analysis, we found that IL21-AS1 and the 3′-UTR of CD24 contain the putative miR-561-5p binding site. Furthermore, miR-561-5p expression was significantly inversely correlated with IL21-AS1 expression. Direct binding of miR-561-5p to IL21-AS1 and to CD24 mRNA was confirmed quite convincingly with a dual-luciferase reporter assay, providing evidence that IL21-AS1 functions as a miR-561-5p sponge to regulate the expression of CD24.

This study showed that IL21-AS1, a lncRNA previously unreported in cancer, was upregulated in OC tissues and that its expression was higher in patients with advanced disease. Functional cellular assays demonstrated that IL21-AS1 could protect cancer cells from macrophage-mediated phagocytosis and promote ovarian tumorigenesis via the IL21-AS1–HIF-1α–CD24 and/or IL21-AS1–miR-561-5p–CD24 axes (Fig. 7J). IL21-AS1, a novel hypoxia-responsive lncRNA, regulates membrane and cytoplasmic CD24 expression and is thus a potential diagnostic and prognostic marker for OC.

### Supplementary information

Supplementary

Figure S1

Figure S2

Figure S3

Figure S4

Table S1

Table S2

Original Western Blot Images

## Data Availability

All data and computer code analyzed in this research are available from the corresponding author on reasonable request. Original western blots images are provided in Supplementary Materials.
