# Supplementary material for: LncRNA IL21-AS1 facilitates tumour progression by enhancing CD24-induced phagocytosis inhibition and tumorigenesis in ovarian cancer
Source: Cell Death Dis. 2024 May 3;15(5):313. doi: 10.1038/s41419-024-06704-8 (PMC11068771; doi:10.1038/s41419-024-06704-8)
Supplement: Supplementary file 1 — Supplementary [file 41419_2024_6704_MOESM1_ESM.docx]

**Supplementary Figures**

**Figure S1**. Knockdown and overexpression efficiencies of IL21-AS1 in OC cell lines. **A**, Knockdown efficiencies of IL21-AS1 in SKOV3 cells. **B-D,** Overexpression efficiencies of IL21-AS1 in SKOV3 (**B**) A2780 (**C**) and ES2 cells (**D**). n = 3, biological replicates. All results are shown as mean ± SD. Statistical significance was accessed by Student’s t test. **P* < 0.05, *****P* < 0.0001.

**Figure S2**. Gating strategy for evaluation of in vivo macrophage-mediated phagocytosis of OC cells, CD24^+^ cancer cells, EdU incorporation assay and apoptosis assay.

**Figure S3**. Localisation of CD24 in OC cells. **A**, Confocal fluorescence micrographs of CD24 in SKOV3, A2780 and ES2 cells. Scale bar, 50 μm. **B**, Flow cytometric analysis of CD24 expression in SKOV3, A2780 and ES2 cells.

**Figure S4**. The CD47 protein level did not change with altered expression of IL21-AS1. **A**, CD47 protein expression in SKOV3 cells transfected with sh-NC or sh-IL21-AS1 plasmids. Left, representative images. Right, statistical analysis. n = 3, biological replicates. **B**, CD47 protein expression in A2780 cells transfected with OE-IL21-AS1 and control plasmids. Left, representative images. Right, statistical analysis. n = 3, biological replicates. All results are shown as mean ± SD. Statistical significance was accessed by Student’s t test. NS not significant.

**Supplementary Tables**

**Table S1**. The clinical characteristics of the ovarian cancer patients.

**Table S2**. The infiltrated immune cells of ovarian cancer tissues in a TCGA cohort based on the IL21-AS1 expression level.
