# Supplementary figures and images for: LncRNA IL21-AS1 facilitates tumour progression by enhancing CD24-induced phagocytosis inhibition and tumorigenesis in ovarian cancer
Source: Cell Death Dis. 2024 May 3;15(5):313. doi: 10.1038/s41419-024-06704-8 (PMC11068771; doi:10.1038/s41419-024-06704-8)

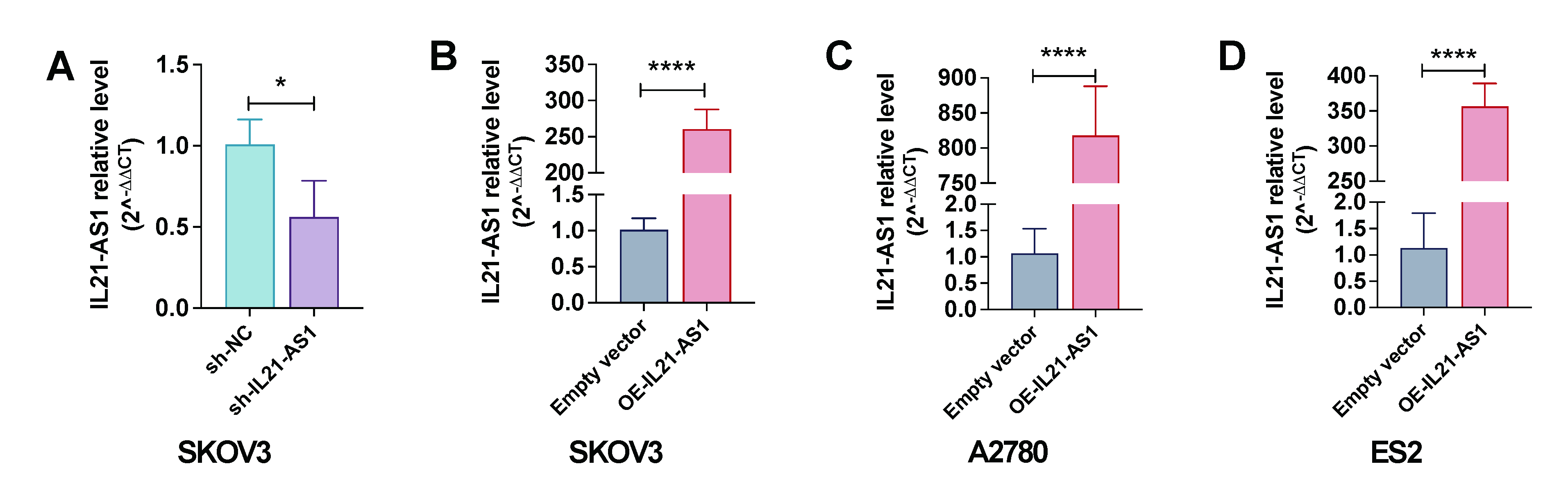

Supplement: Supplementary file 2 — Figure S1 [file 41419_2024_6704_MOESM2_ESM.tif]

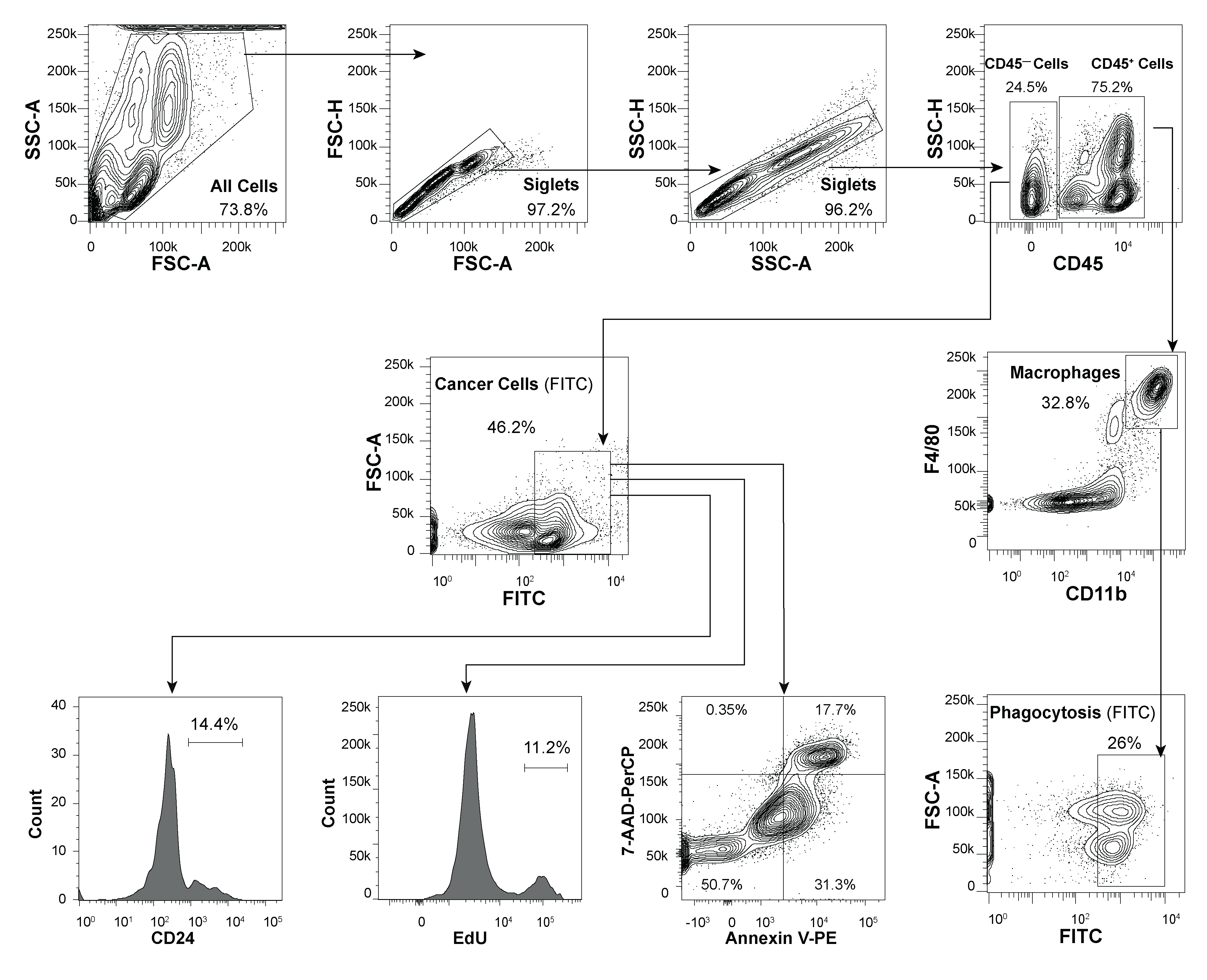

Supplement: Supplementary file 3 — Figure S2 [file 41419_2024_6704_MOESM3_ESM.tif]

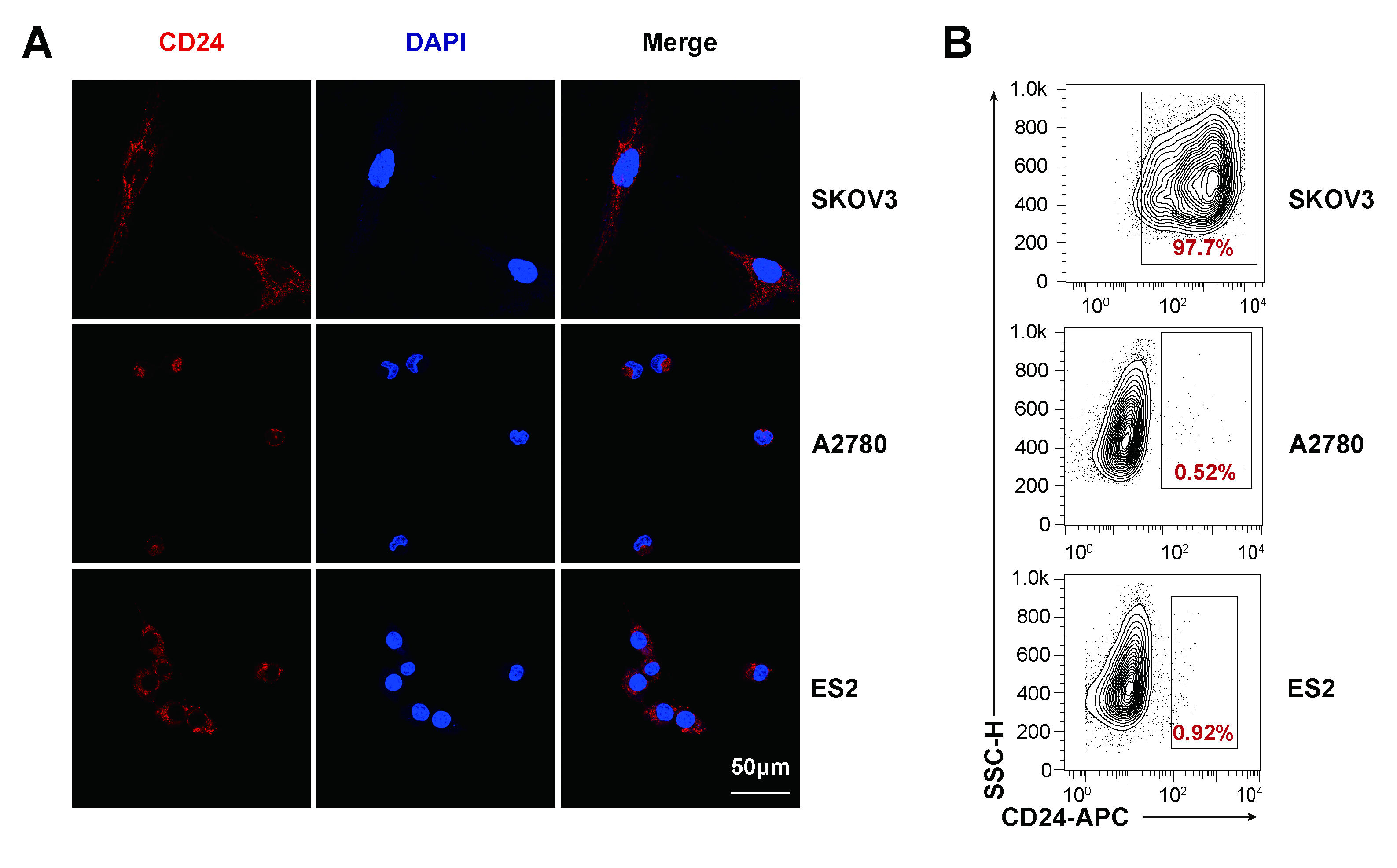

Supplement: Supplementary file 4 — Figure S3 [file 41419_2024_6704_MOESM4_ESM.tif]

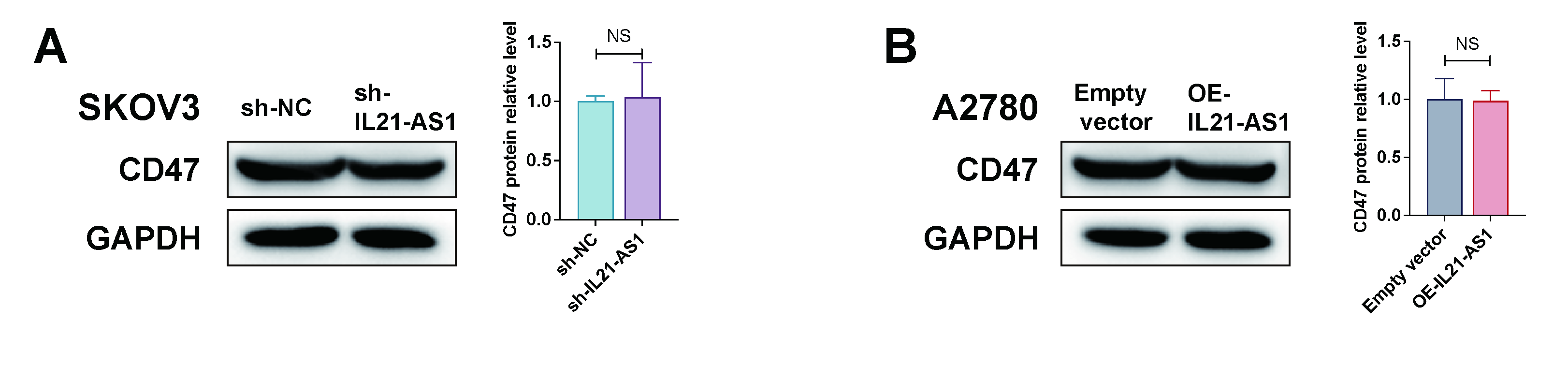

Supplement: Supplementary file 5 — Figure S4 [file 41419_2024_6704_MOESM5_ESM.tif]
