# Supplementary material for: LncRNA IL21-AS1 facilitates tumour progression by enhancing CD24-induced phagocytosis inhibition and tumorigenesis in ovarian cancer
Source: Cell Death Dis. 2024 May 3;15(5):313. doi: 10.1038/s41419-024-06704-8 (PMC11068771; doi:10.1038/s41419-024-06704-8)

Figure 3-D

SKOV3

Cleaved-caspase-3

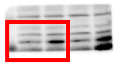

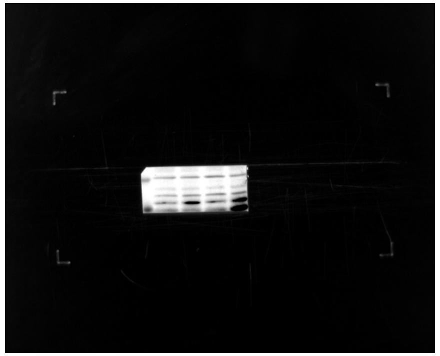

Casepase3

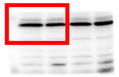

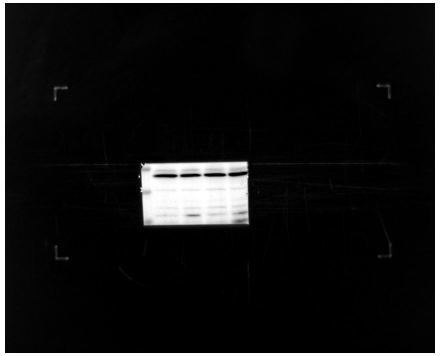

ACTIN

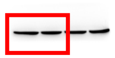

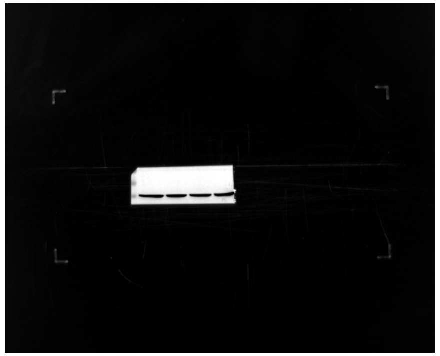

Figure 3-D A2780

Cleaved-caspase-3

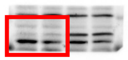

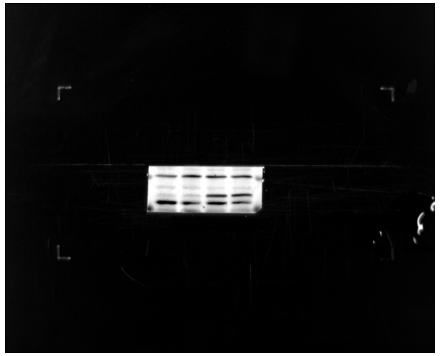

Casepase3

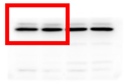

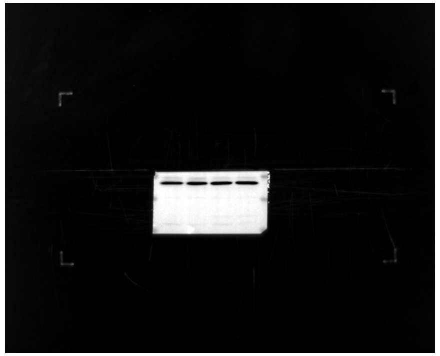

ACTIN

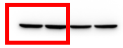

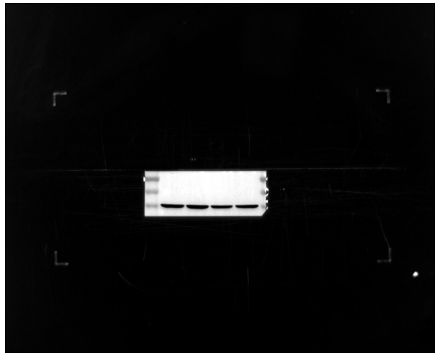

Figure 3-D ES2

Cleaved-caspase-3

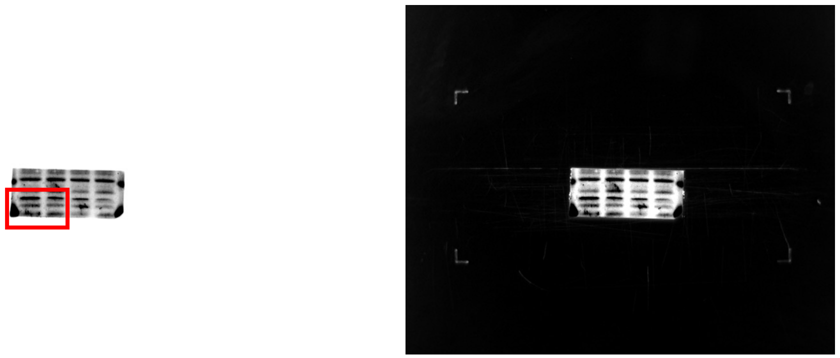

Casepase3

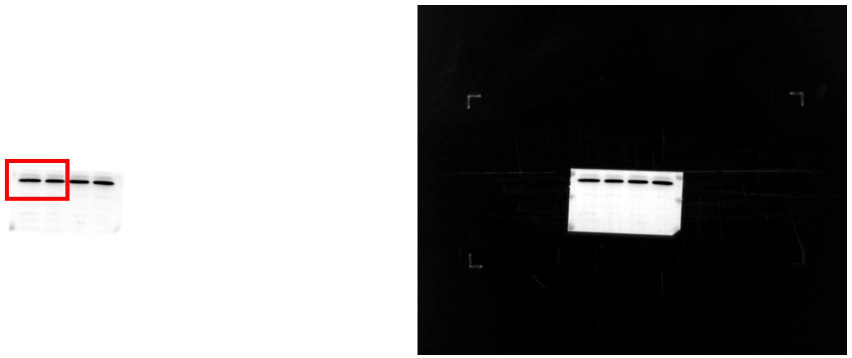

ACTIN

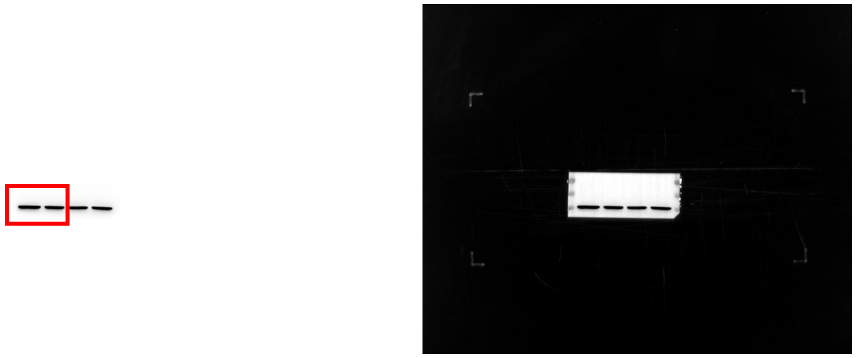

Figure 4-D SKOV3

CD24

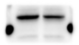

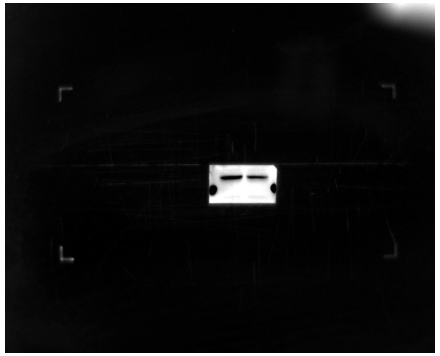

C-Myc

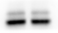

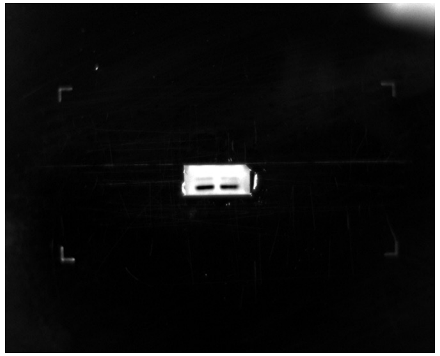

p-STAT3

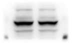

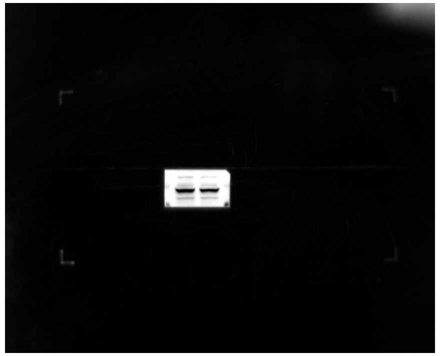

ACTIN

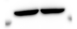

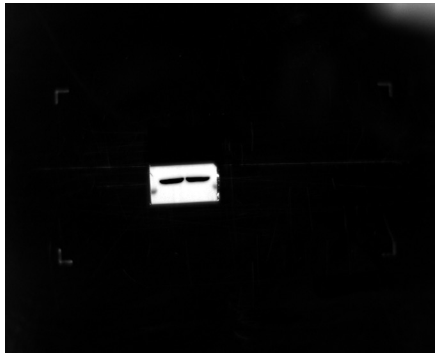

STAT3

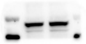

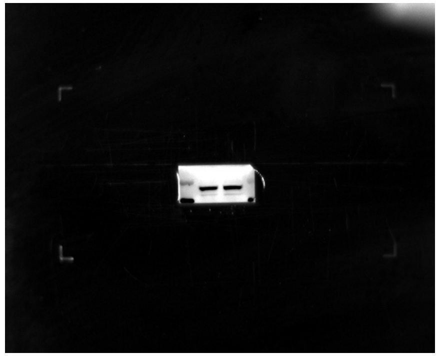

Figure 4-E A2780

CD24

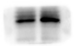

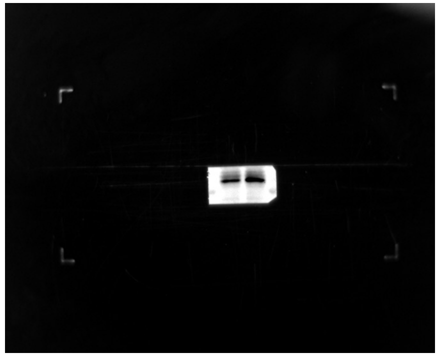

C-Myc

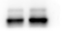

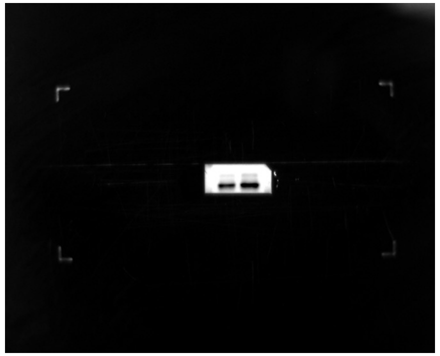

p-STAT3

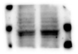

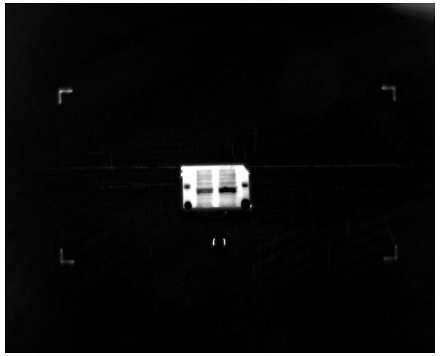

ACTIN

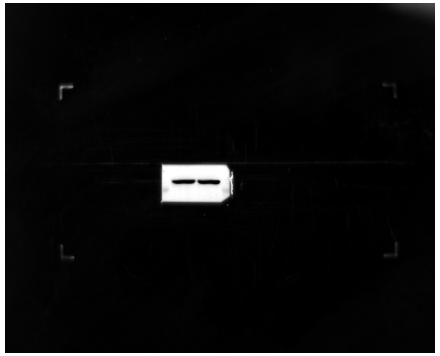

STAT3

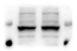

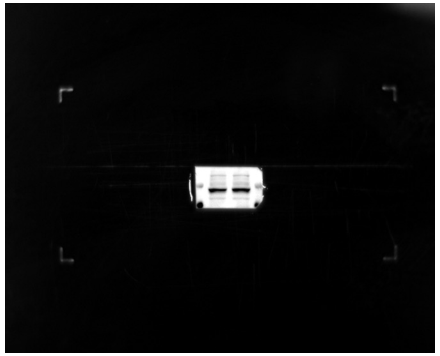

Figure 4-F ES2

CD24

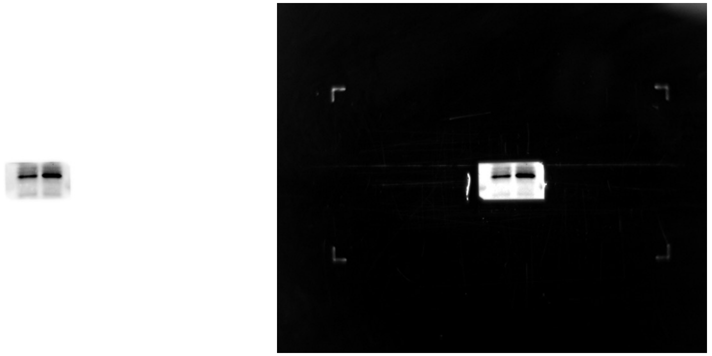

C-Myc

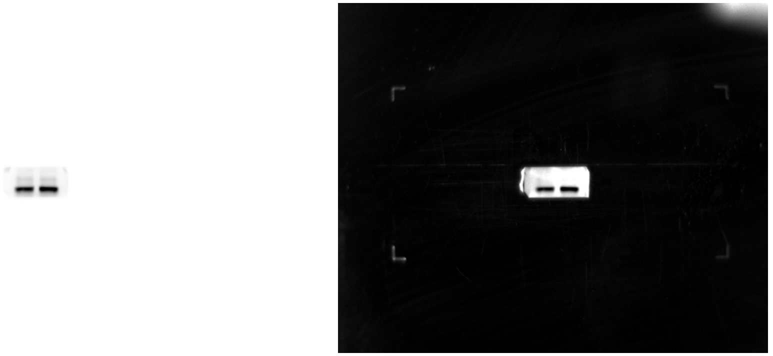

p-STAT3

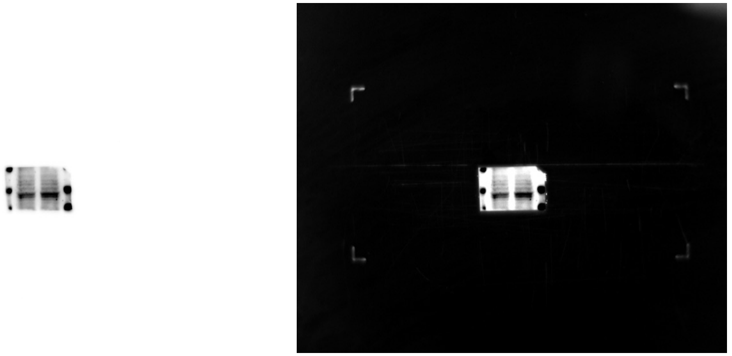

ACTIN

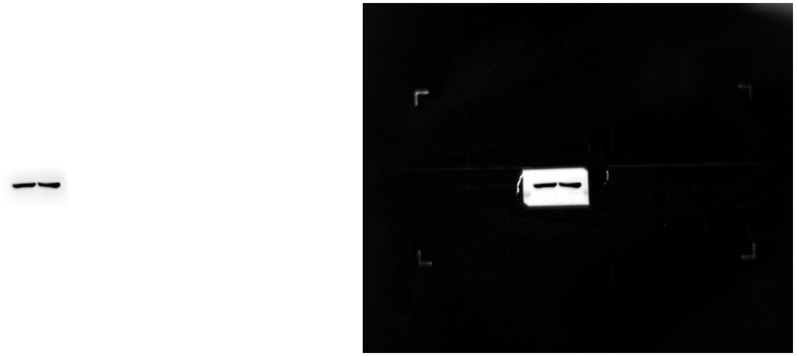

STAT3

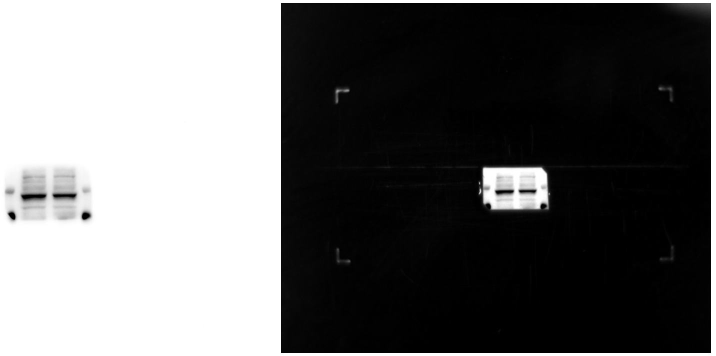

Figure 4-G

CD24

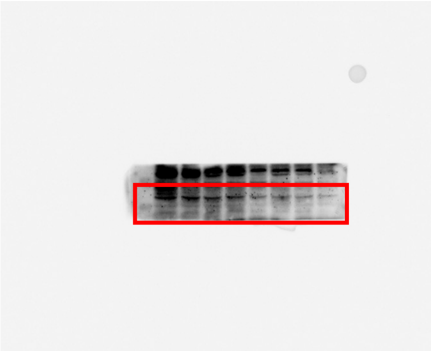

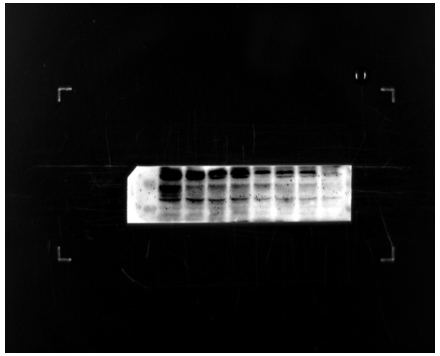

ACTIN

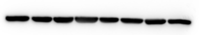

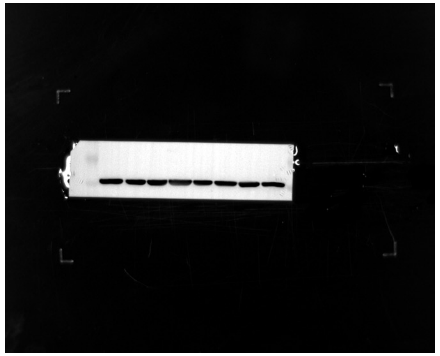

Figure 4-H

CD24

ACTIN

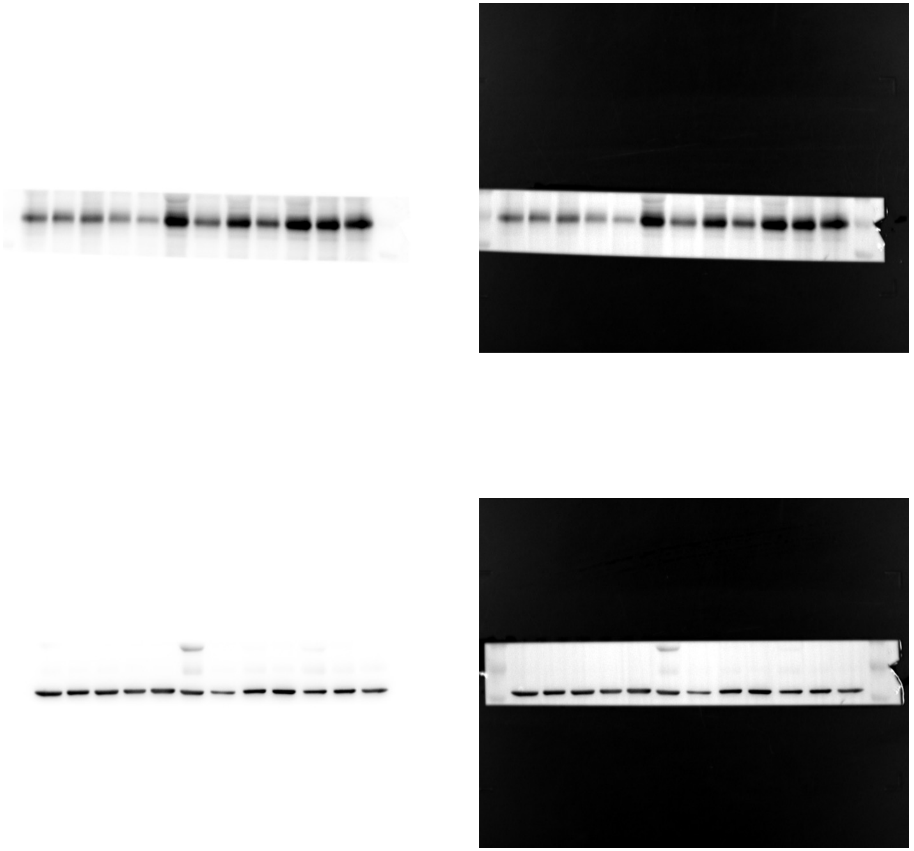

CD24

ACTIN

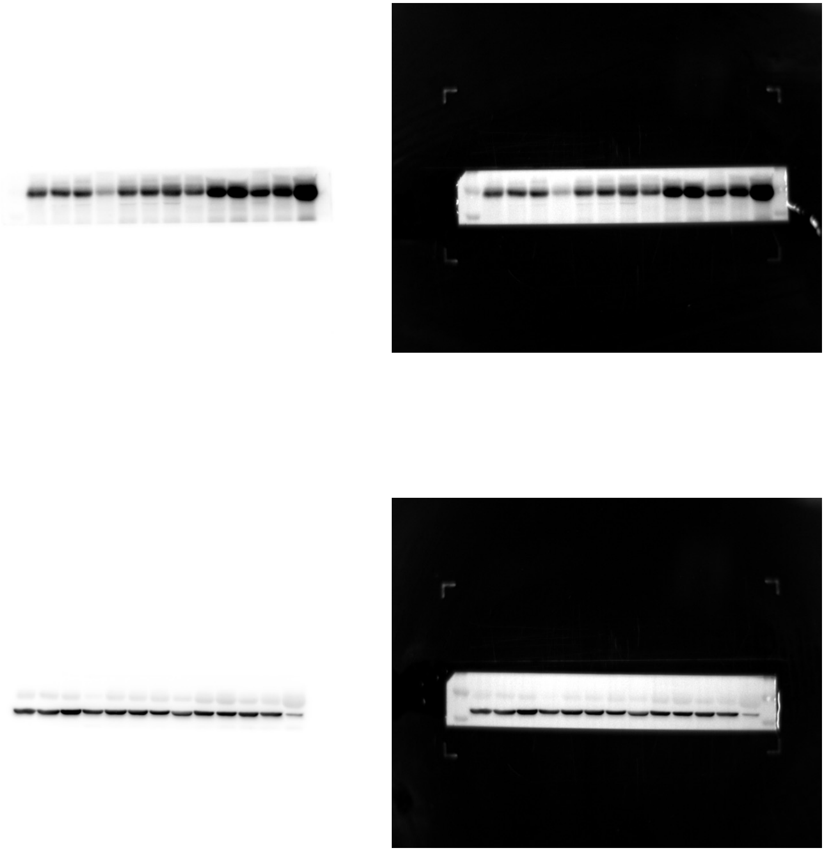

Figure 5-C

HIF-1α

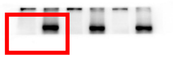

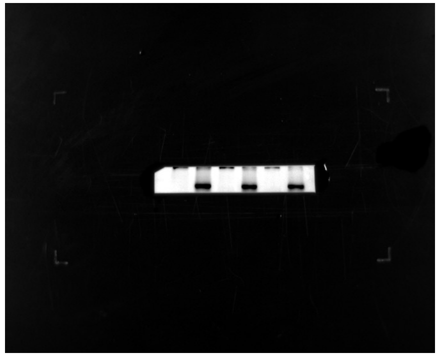

CD24

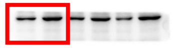

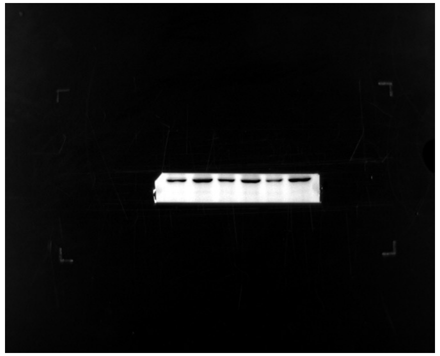

ACTIN

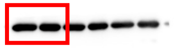

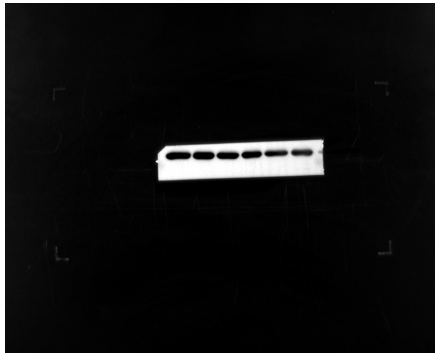

Figure 5-D

HIF-1α

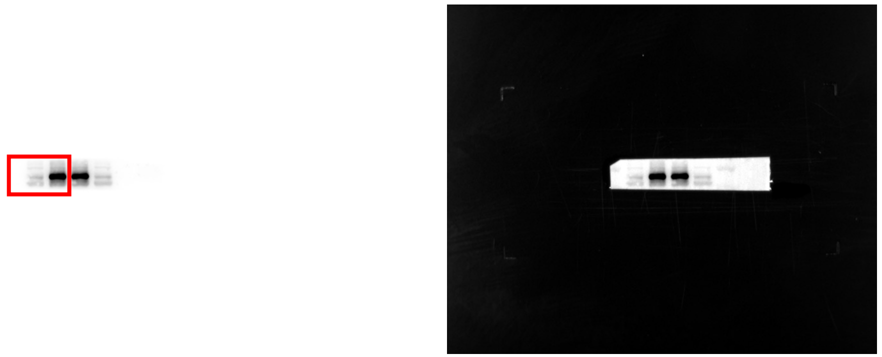

CD24

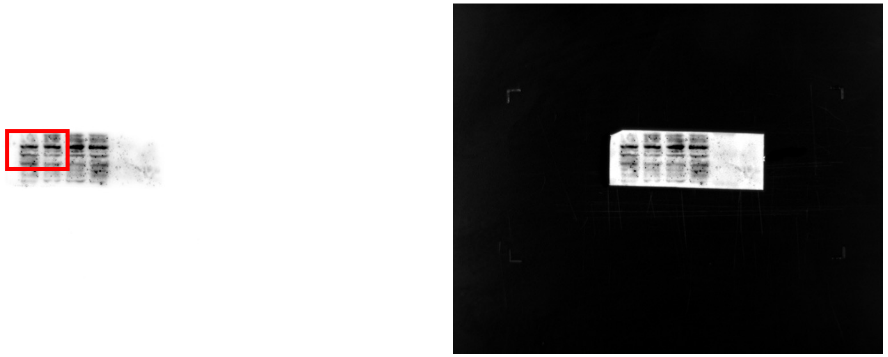

ACTIN

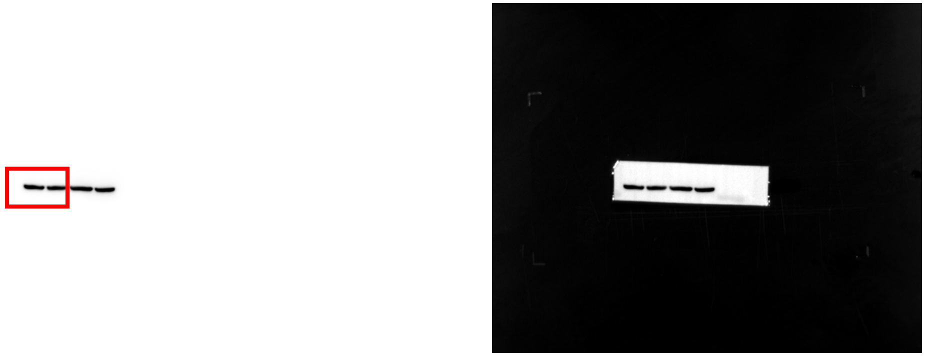

Figure 5-E

HIF-1α

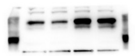

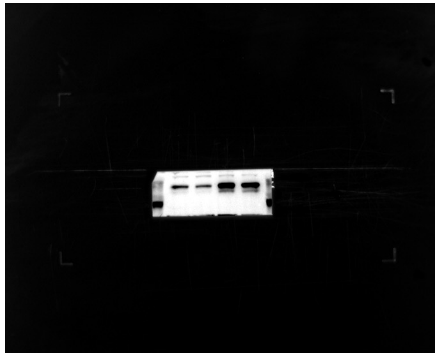

CD24

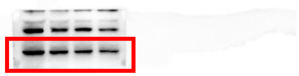

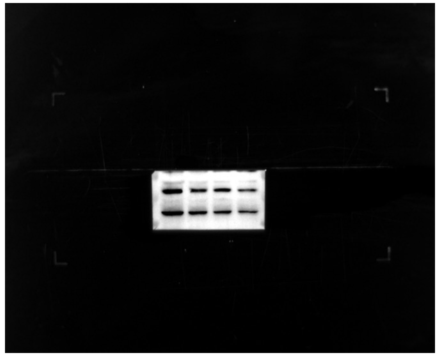

ACTIN

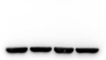

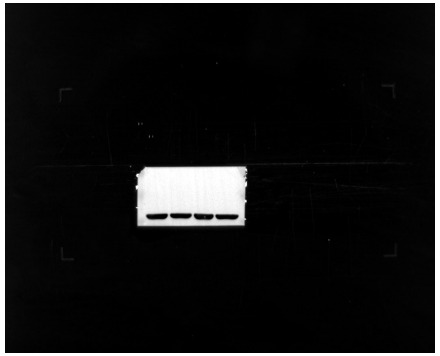

Figure 5-F

HIF-1α

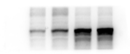

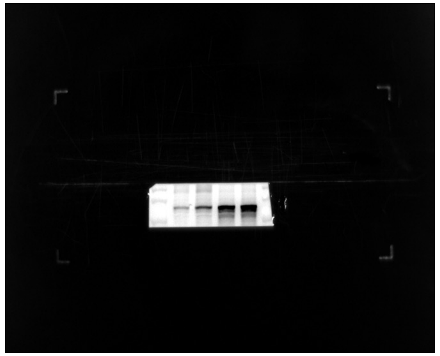

CD24

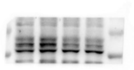

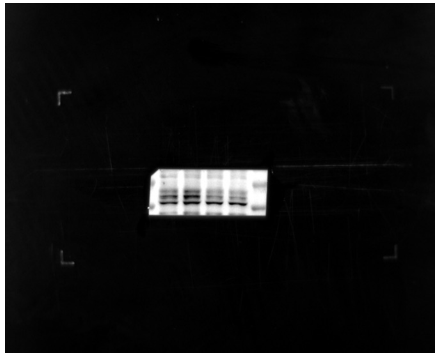

ACTIN

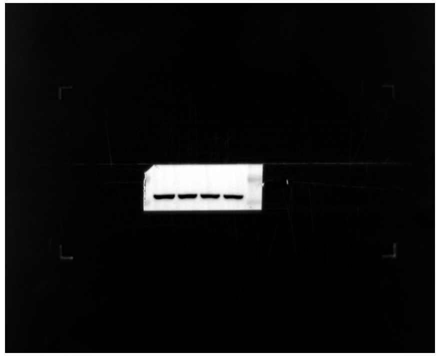

Figure 5-G

HIF-1α

CD24

ACTIN

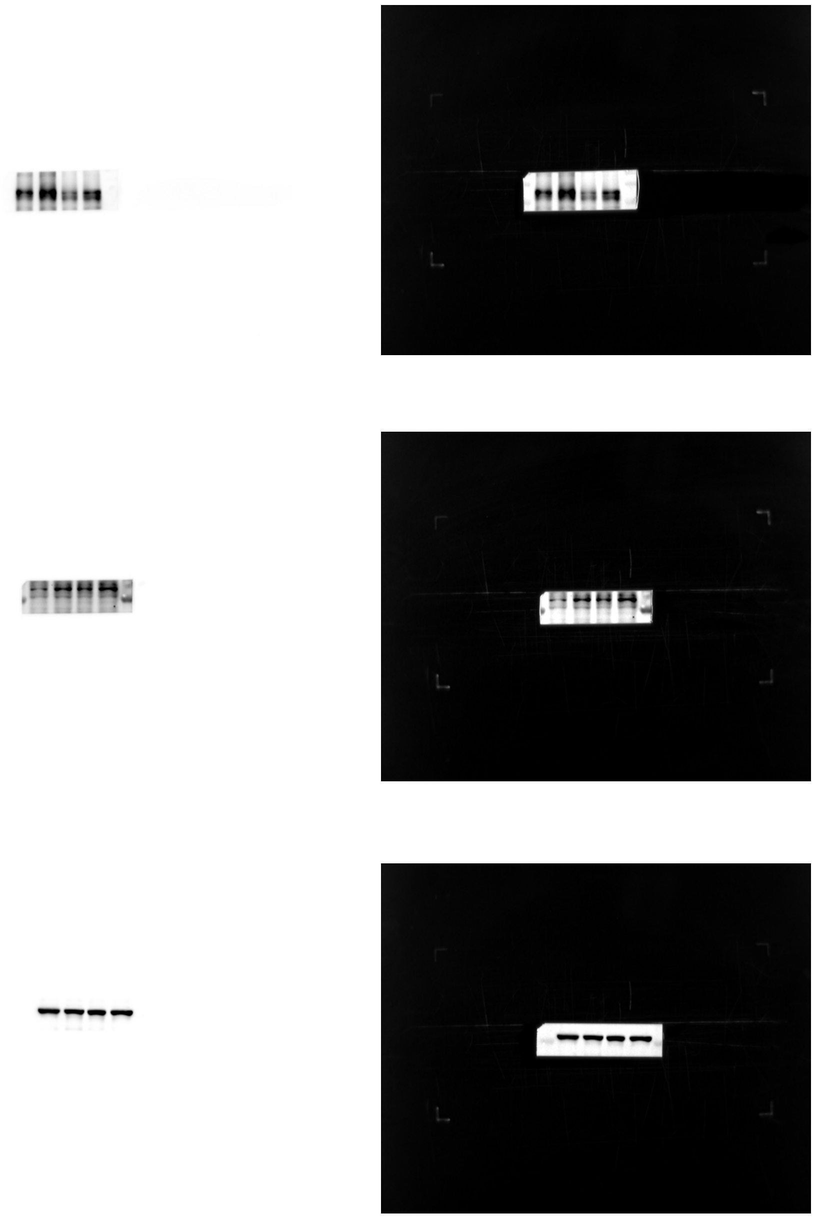

Figure 5-H

HIF-1α

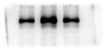

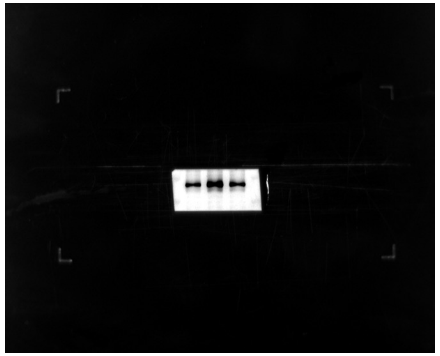

CD24

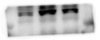

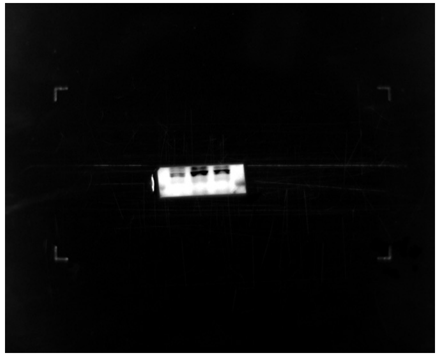

ACTIN

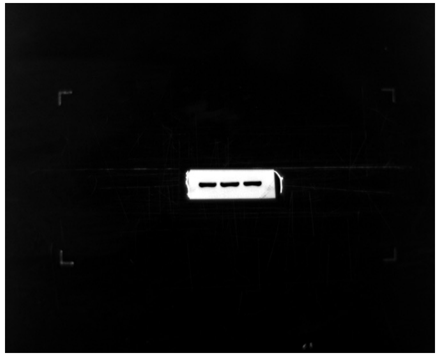

Figure 5-I

HIF-1α

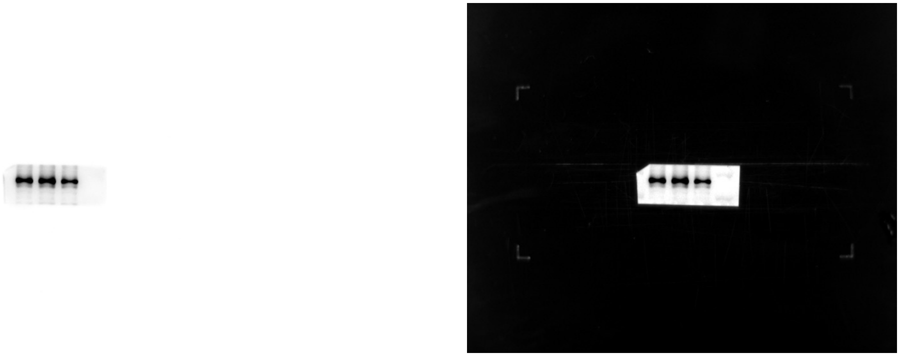

CD24

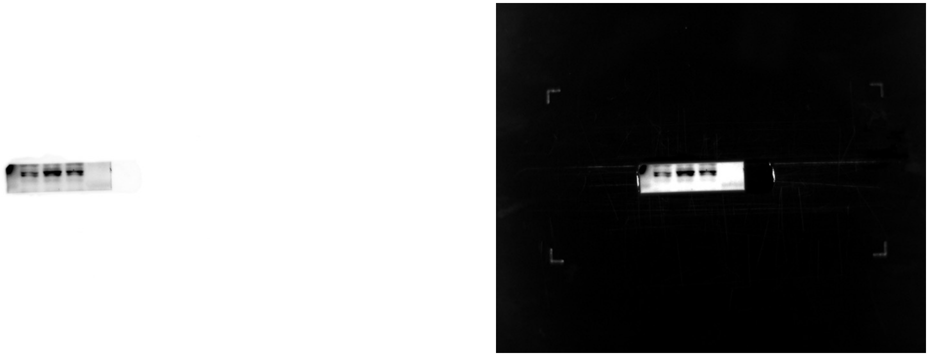

ACTIN

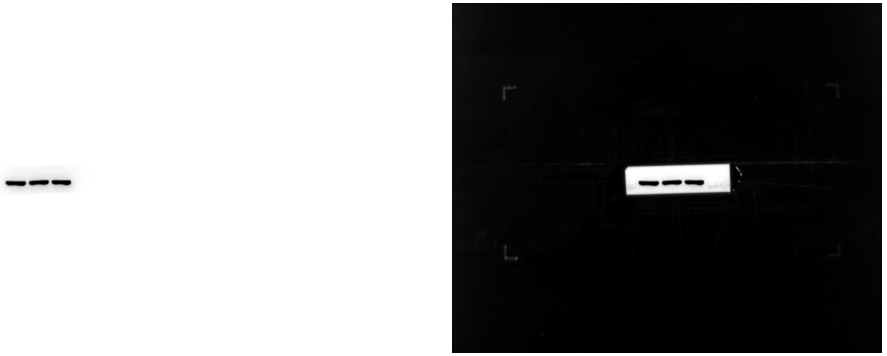

Figure 6-D

CD24

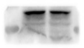

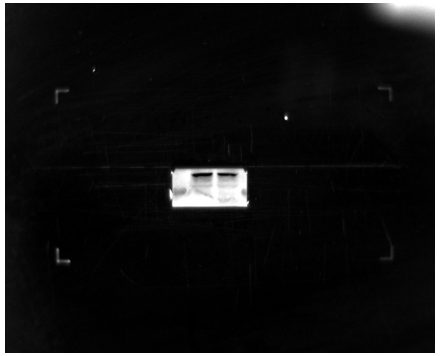

ACTIN

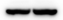

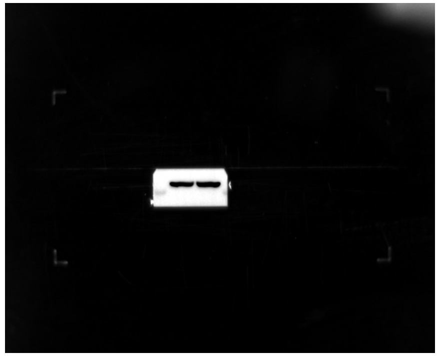

Figure S4-A

CD47

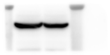

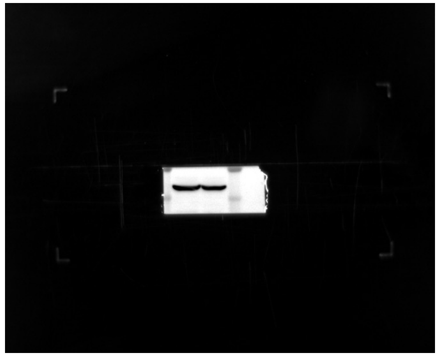

GAPDH

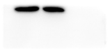

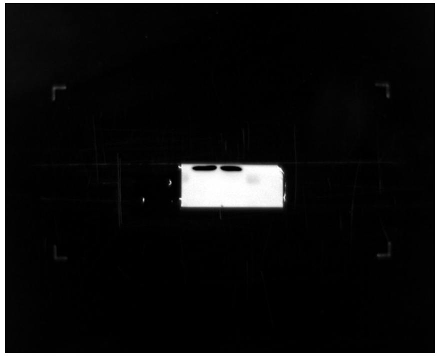

Figure S4-B

CD47

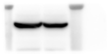

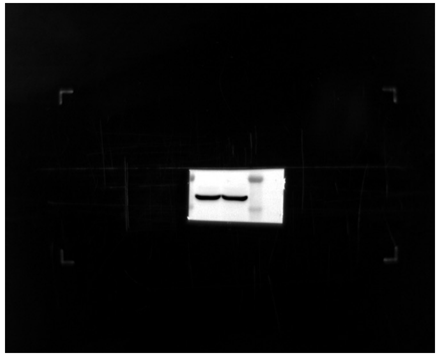

GAPDH

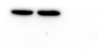

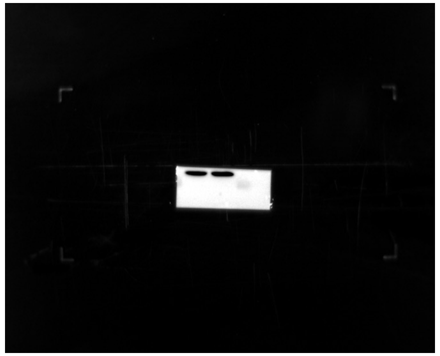

Supplement: Supplementary file 8 — Original Western Blot Images [file 41419_2024_6704_MOESM8_ESM.pdf]
